# The relationship between pregnant women’s attitudes toward preconception care and pregnancy adaptation: a cross-sectional descriptive study

**DOI:** 10.1590/1980-220X-REEUSP-2025-0468en

**Published:** 2026-07-17

**Authors:** Şeyma Çatalgöl, Zehra Üzel

**Affiliations:** 1 Uşak University Faculty of Health Sciences, Department of Nursing, Uşak, Türkiye.

**Keywords:** Preconception Care, Pregnancy, Prenatal Care, Cuidado Pré-Concepcional, Gravidez, Cuidado Pré-Natal

## Abstract

**Objective:**

This study aimed to examine the relationships between pregnant women’s knowledge and attitudes regarding preconception care and their adaptation to pregnancy.

**Method:**

This cross-sectional descriptive study was conducted between 26 December 2023 and 31 July 2024, with 388 pregnant women aged 18–45 who attended the gynecology outpatient clinic of a hospital in the Central Aegean Region of Türkiye. Data were collected using the Introductory Information Form, the Preconception Knowledge and Attitude Scale (PKAS), and the Prenatal Self-Evaluation Questionnaire (PSEQ). Descriptive statistics, Cronbach’s alpha coefficients, and Pearson correlation analysis were used, with significance set at p < 0.05.

**Results:**

Nearly half of the participants (48.5%) received preconception care/counseling, mainly on folic acid and vaccination. Significant negative correlations were found between the two scales and their subscales (p < 0.05). Since lower scores on the PSEQ scale indicate a higher level of adjustment to pregnancy, these findings indicate that higher preconception knowledge and attitudes are associated with better adaptation to pregnancy.

**Conclusion:**

The findings indicate that higher levels of preconception knowledge and attitudes enhance adaptation to pregnancy and underscore the importance of preconception care for a healthy pregnancy and childbirth.

## RESUMO

**Objetivo:**

Este estudo teve como objetivo examinar as relações entre o conhecimento e as atitudes das gestantes em relação aos cuidados pré-concepcionais e sua adaptação à gravidez.

**Método:**

Este estudo descritivo transversal foi realizado entre 26 de dezembro de 2023 e 31 de julho de 2024, com 388 gestantes com idades entre 18 e 45 anos que frequentaram o ambulatório de ginecologia de um hospital na Região do Egeu Central da Turquia. Os dados foram coletados por meio do Formulário de Informações Introdutórias, da Escala de Conhecimento e Atitude Pré-concepcional (PKAS) e do Questionário de Autoavaliação Pré-natal (PSEQ). Foram utilizadas estatísticas descritivas, coeficientes alfa de Cronbach e análise de correlação de Pearson, com significância definida em p < 0,05.

**Resultados:**

Quase metade das participantes (48,5%) recebeu aconselhamento pré-concepcional, principalmente sobre ácido fólico e vacinação. Foram encontradas correlações negativas significativas entre as duas escalas e suas subescalas (p < 0,05). Como pontuações mais baixas na escala PSEQ indicam um maior nível de adaptação à gravidez, essas relações apontam para uma correlação positiva.

**Conclusão:**

Os resultados indicam que níveis mais elevados de conhecimento e atitudes pré-concepcionais melhoram a adaptação à gravidez e ressaltam a importância dos cuidados pré-concepcionais para uma gravidez e um parto saudáveis.

## INTRODUCTION

Preconception care/counseling services is a crucial preventive health service that encompasses comprehensive interventions to optimize health before pregnancy, aiming to improve pregnancy rates, pregnancy outcomes, infant health, and the health of future generations^(1)^.

Preconception care/counseling is a primary prevention approach that aims to identify potential health risks for women and reproductive-aged couples before conception, as well as to provide a range of education and management interventions to address these risks. Risks include biological, environmental and behavioral factors. In this context, genetic disorders, teratogen exposure, lifestyle habits (smoking, substance use, diet), and folic acid deficiency are prominent^(2)^.

Preconception care/counseling is particularly important for patients with high-risk conditions, which can have significant impacts early in the first trimester, before most women begin prenatal care. For women who choose to postpone pregnancy, preconception care/counseling offers an opportunity to provide contraceptive care^(3)^.

Some studies have found evidence that exposure to teratogens or environmental conditions that may affect fertility in the preconception period impacts physical, mental, social, and psychological health and increases the risk of preeclampsia, gestational diabetes, and adverse fetal outcomes^(4,5)^.

A systematic review found that interventions such as delaying the age at first pregnancy and optimizing the interval between pregnancies, which are among the preconception care/counseling practices, increased contraceptive use. Furthermore, the use of folic acid-iron combinations during the preconception period has been reported to reduce anemia, and folic acid use has been reported to reduce the incidence of neural tube defects^(6)^.

A woman’s pregnancy experience throughout her life influences various aspects of her lifestyle and leads to changes in her social role. This period is a period of psychosocial adjustment for women, during which they may experience uncertainties about pregnancy and motherhood^(7)^. Women with physical and mental health problems during the preconception period are at greater risk during pregnancy compared to women without such problems, and their adaptation to pregnancy is more difficult^(8)^.

Adapting to pregnancy is the process of making sense of the mother’s physical and psychosocial changes and is influenced by factors such as family support, health status, and pregnancy complications^(9)^. High-risk pregnancies and health problems can complicate adaptation to pregnancy and increase women’s stress and anxiety levels^(10)^. Therefore, midwives can facilitate women’s adaptation to pregnancy and better prepare them for motherhood through the support they provide to pregnant women and positive pregnancy experiences^(11)^.

Preparing women for pregnancy from the preconception period, identifying and treating existing risk conditions before conception, providing preventive health services, and preparing them psychologically and physically for pregnancy can all facilitate women’s adjustment to pregnancy and motherhood.

While preconceptional care/counseling has significant impacts on maternal and infant health, there is limited evidence regarding the relationship between Turkish pregnant women’s knowledge and attitudes towards preconceptional care and their adjustment to pregnancy. This knowledge gap is particularly important for understanding the prevalence and impact of preconceptional care/counseling services. The present research examines the relationship between preconception knowledge and attitudes and adaptation to pregnancy among pregnant women in Türkiye. In this context, two hypotheses have been put forward: Hypothesis 1 predicts that women who received preconceptional care/counseling would have higher levels of adjustment to pregnancy than those who did not. Hypothesis 2 predicts that women with more knowledge about preconceptional care would have higher levels of adjustment to pregnancy than those with less knowledge.

## METHOD

### Study Design

This study is a cross-sectional, descriptive study. The study population consisted of all pregnant women who presented to the Obstetrics and Gynecology Outpatient Clinic of Uşak Training and Research Hospital between 26 December 2023 and 31 July 2024 and met the study criteria. Reporting of the study was conducted in accordance with the STROBE guidelines^(12)^.

### Inclusion Criteria

- Pregnant women aged 18–45 years,
- Between 6 and 42 weeks of gestation,
- Who voluntarily agreed to participate in the study during the specified dates.

### Exclusion Criteria

- Cognitive impairments or serious health conditions that would prevent reliable participation,
- Pregnancies complicated by serious medical conditions requiring specialist intervention,
- Complications, intercurrent events, or pathologies that existed before pregnancy or arose during pregnancy.

### Study Sample

The sample size was calculated using OpenEpi software and the known population formula n = [DEFF × N × p × (1–p)]/[(d^2^/Z^2^_1−_α/_2_ × (N–1)) + p × (1–p)]. According to the Turkish Statistical Institute (TÜİK, 2022), the female population aged 18–45 in Türkiye was estimated at approximately 17.8 million based on age-specific population data from the Address-Based Population Registration System as of December 31, 2022. The minimum required sample size was calculated to be 385 participants. Considering a potential 10% data loss, the target sample size was set at 428 participants. To achieve this target, 449 pregnant women were approached, of whom 428 agreed to participate in the study. After excluding 40 participants with missing data, the final analysis was conducted on 388 participants.

### Data Collection Tools

Pregnant women who met the study criteria and agreed to participate after being informed about the study were given an introductory information form, and the Preconception Knowledge and Attitude Scale (PKAS) along with the Prenatal Self-Evaluation Questionnaire (PSEQ) were administered. Data were collected by the researchers using a face-to-face data collection method.

### Descriptive Information Form

This form was developed by the researchers to determine the socio-demographic and obstetric characteristics of the pregnant women. The socio-demographic characteristics included age, education level, marital status, consanguineous marriage, occupation, social security, chronic disease, smoking, alcohol use. The obstetric characteristics section consisted of 23 questions to assess the number of pregnancies, obstetric history, and preconceptional care receipt.

### Preconceptional Knowledge and Attitude Scale (PKAS)

The scale, developed by Gokdemir and Eryilmaz^(14)^, consists of 43 items and 7 subscales The subscales are:

1. Attitudes Towards of Preconceptional Health Protection and Improving
2. Attitudes Towards of Planning Pregnancy
3. Attitudes Toward of Previous Life Conditions Before Having a Child
4. Attitudes Towards of Preconceptional Risk Factors
5. Attitudes Towards of Preconceptional Health Behaviors
6. Behaviors that should be Avoided in the Preconceptional Period
7. Related to Preconception Health Sensitivity Status.

Subdimension names are presented as in the original scale. It consists of a total of 43 items, 42 positive and one negative. The 12th item of the scale (“If my marriage goes bad, I will have a child to save my marriage”) is reverse scored. The scale is a 5-point Likert-type scale, and higher scores indicate more positive preconception knowledge and attitudes. The reliability (Cronbach’s Alpha) coefficient of the scale was reported as 0.86, and the test-retest correlation coefficient was found to be r = 0.978.

### Prenatal Self Evaluation Questionnaire (PSEQ)

This questionnaire is a 79-item, 4-point Likert-type scale developed by Lederman in 1979 to assess women’s adjustment to motherhood during the prenatal period. The validity and reliability of the scale in Türkiye were assessed by Beydağ and Mete, and Cronbach’s alpha was found to be 0.81. The scale has seven subdimensions: Concern for the Well-Being of Self and Baby, Acceptance of Pregnancy, Identification of a Motherhood Role, Preparation for Labour, Fear of Helplessness and Loss of Control in Labour, Relationship with Her Mother, Relationship with Her Husband/Partner. Forty-seven of the scale’s items are reverse-coded. This scale is evaluated based on scores ranging from 1 to 4 (4: Describes very well, 3: Describes somewhat, 2: Describes a little, 1: Does not describe at all). For reverse-coded items, the scoring is done in the opposite direction. The total scale score can range from a minimum of 79 to a maximum of 316. Lower scores indicate a higher level of adaptation to pregnancy^(15,16)^. Therefore, negative values in correlation analyses indicate a positive association with greater adaptation to pregnancy.

### Variables

#### Independent Variables

Sociodemographic characteristics (age, education level, marital status, consanguineous marriage, occupation, social security, chronic disease, smoking, alcohol use), obstetric history (number of pregnancies and births, history of stillbirth, and complications experienced in previous pregnancies or births), pregnancy-related factors (whether the current pregnancy is planned and whether preconceptional care/counseling was received), and preconceptional knowledge and attitude levels measured by PSEQ (total and subscale scores) were included as independent variables.

#### Dependent Variable

The outcome variable was adherence to pregnancy, assessed using PSEQ total and subscale scores.

#### Confounding Variables

Age, education level, consanguineous marriage, occupation, social security status, chronic disease status, smoking, history of stillbirth, presence of complications threatening the previous pregnancy, presence of complications in previous childbirth, planned status of the current pregnancy, receipt of care and counseling services for the current pregnancy during the preconception period were considered as potential confounding variables.

### Data Analysis

The Statistical Package for Social Sciences for Windows 25.0 (IBM Corp., Armonk, NY, USA) software was used to analyze the data. Descriptive statistics for the data used in the study were presented as mean and standard deviation for continuous variables, and as median, minimum, maximum, and percentage (%). Furthermore, for variables where parametric and especially nonparametric tests were applied, descriptive statistics were presented in tables as mean-standard deviation and median (min-max). A significance level of p < 0.05 was adopted to determine whether there was a statistically significant difference between the groups. When comparing two independent groups, the independent samples t-test was used when the continuous variables followed a normal distribution, and the Mann–Whitney U test was used when the assumption of normal distribution was not met. One-way analysis of variance (ANOVA) was used to compare three or more independent groups that followed a normal distribution. When the assumptions were not met, the Kruskal–Wallis H test was applied. The chi-square test was used to compare demographic variables, categorical variables, and scale and scale subscales. Pearson correlation analysis was used to determine the relationship between variables when a normal distribution was achieved, and Spearman correlation analysis was used when a normal distribution was not achieved.

### Ethical Aspects

This study was conducted in accordance with the principles of the Declaration of Helsinki. Ethics committee approval was obtained from the Uşak University Non-Interventional Clinical Research Ethics Committee (Date: December 14, 2023; Decision No: 267-267-34). Institutional permission for the study was obtained from the relevant Research Hospital (Date: November 22, 2023; Number: E45786011-599-230168660).

## RESULTS

Among the 449 pregnant women approached for participation, 21 declined to participate and 428 were enrolled in the study. Following the exclusion of 40 participants with missing data, the final analytical sample comprised 388 participants.

### Socio-Demographic Characteristics of Participants

The sociodemographic characteristics of the participants are presented in Table 1. It was observed that 46.6% of the participants were between the ages of 24–29, 44.8% were university graduates, 99.2% were married, and 7% had consanguineous marriages. Additionally, 57.7% were homemaker, 84.3% had social security coverage, 8% reported having a chronic disease (these included manageable conditions not requiring specialized intervention, e.g., mild asthma, controlled hypothyroidism, diet-controlled diabetes), 10.6% smoked during their current pregnancy, and 0.8% consumed alcohol during pregnancy (Table 1).

**Table 1:** Participants’ socio-demographic characteristics – Uşak, TR, Türkiye, 2024.

Variables	n	%
**Age**		
18–23	71	18.3
24–29	181	46.6
30–35	103	26.5
36–45	33	8.5
**Educational status**		
Primary school or below	50	12.9
Secondary school	146	37.6
College/University graduate	174	44.8
Postgraduate (Master’s/Doctorate)	18	4.6
**Marital status**		
Married	385	99.2
Single	3	0.8
**Consanguineous marriage status**		
Yes	27	7.0
No	361	93.0
**Occupation**		
Homemaker	224	57.7
Civil servant	93	24.0
Worker	46	11.9
Self-employed	25	6.4
**Social security status**		
Yes	327	84.3
No	61	15.7
* **Chronic disease status**		
Yes	31	8.0
No	357	92.0
**Smoking status**		
Currently smoking	41	10.6
Never smoked	292	75.3
Quit due to pregnancy	55	14.2
**Alcohol consumption status**		
Currently using	3	0.8
Never used	342	88.1
Quit due to pregnancy	43	11.1
**Total**	**388**	**100.0**

*Chronic diseases included manageable conditions not requiring specialized intervention (e.g., mild asthma, controlled hypothyroidism, diet-controlled diabetes).

Obstetric Characteristics and Preconception Care Status of Participants

The participants’ obstetric characteristics and preconception care status are presented in Table 2. Half of the participants (50%) were experiencing their first pregnancy (primigravida). Among the 194 multigravida women, 12.4% reported a history of miscarriage, 8% reported a history of stillbirth, 10.3% experienced a complication that posed a risk to the pregnancy, and 9% reported experiencing complications during labor. Overall, 73.2% of participants stated that their current pregnancy was planned (Table 2).

**Table 2:** Participants’ obstetric characteristics and preconception care status – Uşak, TR, Türkiye, 2024.

Variables	n	%
**Number of pregnancies**		
Primigravida	194	50
Multigravida	194	50
**History of miscarriage**		
Yes	48	12.4
No	340	87.6
**History of stillbirth**		
Yes	31	8
No	357	92
**Number of living children**		
Nullipara	212	54.7
Primipara	104	26.8
Multipara	72	18.5
**Presence of complications threatening the previous pregnancy**		
Yes	40	10.3
No	154	39.7
Primigravida	194	50
**Presence of complications in previous childbirth**		
Yes	35	9.0
No	159	41
Primigravida	194	50
**Planned status of the current pregnancy**		
Yes	284	73.2
No	104	26.8
**Receipt of care/counseling services for the current pregnancy during the preconception period**		
Yes	188	48.5
No	200	51.5
**Topics covered during preconception counseling Smoking cessation**		
Yes	12	3.1
No	376	96.9
**Alcohol cessation**		
Yes	6	1.5
No	382	98.5
**Folic acid/vitamin use**		
Yes	117	30.2
No	271	69.8
**Vaccination status**		
Yes	84	21.6
No	304	78.4
**Treatment and management of chronic diseases**		
Yes	25	6.4
No	363	93.6
**Adequate and balanced nutrition**		
Yes	79	20.4
No	309	79.6
**Weight management**		
Yes	64	16.5
No	324	83.5
**Genetic inherited diseases**		
Yes	20	5.2
No	368	94.8
**Consanguineous marriage**		
Yes	7	1.8
No	381	98.2
**Other**		
Yes	2	0.5
No	386	99.5
**Total**	**388**	**100.0**

It was found that 48.5% of the participants received care/counseling services in the preconception period for their current pregnancy. The most frequently addressed topics included folic acid/vitamin use (30.2%), vaccination (21.6%), adequate and balanced nutrition (20.4%), and weight management (16.5%). Other topics included chronic disease management (6.4%), hereditary diseases (5.2%), smoking cessation (3.1%), consanguineous marriage (1.8%), alcohol cessation (1.5%), and other subjects (0.5%).

### Scale Scores and Reliability of Participants

Descriptive statistics and reliability coefficients of the scales used in the study are presented in Table 3. The mean score for the PKAS was 194.32 ± 16.76 (Cronbach’s α = 0.901), and the mean score for the PSEQ was 133.46 ± 27.02 (Cronbach’s α = 0.922). The internal consistency of both scales, including their subdimensions, was found to be high (Table 3).

**Table 3:** Descriptive statistics of PKAS and PSEQ scores – Uşak, TR, Türkiye, 2024.

Scales and their dimensions	Min	Max	Mean	SD	Cronbach’s Alpha
**Preconception Care Knowledge and Attitude Scale**	141.00	215.00	194.32	16.76	0.901
Attitudes Towards of Preconceptional Health Protection and Improving	14.00	50.00	46.63	4.29	0.746
Attitudes Towards of Planning Pregnancy	10.00	35.00	31.55	3.64	0.749
Attitudes Toward of Previous Life Conditions Before Having a Child	9.00	25.00	21.41	3.17	0.812
Attitudes Towards of Preconceptional Risk Factors	15.00	40.00	36.25	4.09	0.729
Attitudes Towards of Preconceptional Health Behaviors	10.00	45.00	21.48	3.74	0.813
Behaviors that should be Avoided in the Preconceptional Period	9.00	25.00	22.81	2.83	0.811
Related to Preconception Health Sensitivity Status	4.00	15.00	14.19	1.49	0.799
**Prenatal Self Evaluation Questionnaire**	83.00	230.00	133.46	27.02	0.922
Concern for the Well-Being of Self and Baby	10.00	38.00	22.09	6.30	0.806
Acceptance of pregnancy	14.00	62.00	21.37	5.94	0.797
Identification of a Motherhood Role	15.00	44.00	23.04	5.36	0.823
Preparation for Labour	10.00	47.00	17.49	4.63	0.744
Fear of Helplessness and Loss of Control in Labour	10.00	34.00	20.14	4.62	0.873
Relationship with Her Mother	9.00	37.00	14.51	5.44	0.870
Relationship with Her Husband/Partner	10.00	40.00	14.82	5.23	0.828

Min: Minimum; Max: Maximum; SD: Standard Deviation.

### Correlations between PSEQ and PKAS Scores and Subscales

The relationship between the PSEQ and its subscales and the PKAS and its subscales is presented in Table 4. A statistically significant negative correlation was identified between the total scores of the PSEQ and the PKAS (r = −0.428, p < 0.001, moderate) (Table 4).

**Table 4:** Correlations between PKAS and PSEQ scores and their subscales – Uşak, TR, Türkiye, 2024.

Scales and Dimensions		Prenatal self-evaluation scale	Concern for the well being of self and baby	Acceptance of pregnancy	Identification of a motherhood role	Preparation for labour	Fear of helplessness and loss of control in labour	Relationship with her mother	Relationship with her Husband/Partner
Preconception Care Knowledge and Attitude Scale	r	-0.428**	-0.177**	-0.330**	-0.306**	-0.436**	-0.345**	-0.373**	-0.372**
	p	0.000	0.000	0.000	0.000	0.000	0.000	0.000	0.000
Attitudes Towards of Preconceptional Health	r	-0.376**	-0.177**	-0.257**	-0.272**	-0.321**	-0.296**	-0.303**	-0.365**
	p	0.000	0.000	0.000	0.000	0.000	0.000	0.000	0.000
**Protection and improving**									
Attitudes Towards of Planning Pregnancy	r	-0.337**	-0.155**	-0.273**	-0.258**	-0.268**	-0.230**	-0.294**	-0.345**
	p	0.000	0.002	0.000	0.000	0.000	0.000	0.000	0.000
Attitudes Toward of Previous Life Conditions	r	-0.242**	-0.060	-0.194**	-0.135**	-0.282**	-0.200**	-0.254**	-0.209**
	p	0.000	0.240	0.000	0.008	0.000	0.000	0.000	0.000
**Before Having a Child**									
Attitudes Towards of Preconceptional Risk Factors	r	-0.288**	-0.085	-0.201**	-0.161**	-0.305**	-0.234**	-0.280**	-0.281**
	p	0.000	0.094	0.000	0.001	0.000	0.000	0.000	0.000
Attitudes Towards of Preconceptional Health Behaviors	r	-0.313**	-0.065	-0.231**	-0.229**	-0.383**	-0.289**	-0.333**	-0.264**
	p	0.000	0.205	0.000	0.000	0.000	0.000	0.000	0.000
Behaviors that should be Avoided in the Preconceptional Period	r	-0.300**	-0.102*	-0.221**	-0.235**	-0.328**	-0.258**	-0.301**	-0.201**
	p	0.000	0.044	0.000	0.000	0.000	0.000	0.000	0.000
Related to Preconception Health Sensitivity Status	r	-0.288**	-0.093	-0.201**	-0.185**	-0.321**	-0.198**	-0.279**	-0.313**
	p	0.000	0.067	0.000	0.000	0.000	0.000	0.000	0.000

*p < 0.05;

**p < 0.01;

r: Pearson correlation analysis.

In addition, statistically significant negative correlations were found between the total PSEQ score and the subdimensions of the PKAS. These correlations included: Attitudes Towards of Preconceptional Health Protection and Improving (r = −0.376, p < 0.001, moderate), Attitudes Towards of Planning Pregnancy (r = −0.337, p < 0.001, moderate), Attitudes Toward of Previous Life Conditions Before Having a Child (r = −0.242, p < 0.001, weak), Attitudes Towards of Preconceptional Risk Factors (r = −0.288, p < 0.001, weak), Attitudes Towards of Preconceptional Health Behaviors (r = −0.313, p < 0.001, moderate), Behaviors that should be Avoided in the Preconceptional Period (r = −0.300, p < 0.001, moderate), and Related to Preconception Health Sensitivity Status (r = −0.288, p < 0.001, weak).

Furthermore, significant negative correlations were observed between the total PKAS score and the subdimensions of the PSEQ. These ranged from weak to moderate, including: Concern for the Well-Being of Self and Baby (r = −0.177, p < 0.001, weak), Acceptance of Pregnancy (r = −0.330, p < 0.001, moderate), Identification of a Motherhood Role (r = −0.306, p < 0.001, moderate), Preparation for Labour (r = −0.436, p < 0.001, moderate), Fear of Helplessness and Loss of Control in Labour (r = −0.345, p < 0.001, moderate), Relationship with Her Mother (r = −0.373, p < 0.001, moderate), and Relationship with Her Husband/Partner (r = −0.372, p < 0.001, moderate).

### Relationship between Pregnancy Planning, Preconception Care/Counseling, and Participants’ PKAS and PSEQ Scores

The relationship between pregnancy planning, preconception care/counseling services, and participants’ PKAS and PSEQ scores is presented in Table 5. PKAS scores showed significant differences in terms of planned pregnancy (p = 0.008) and receiving preconception care/counseling (p = 0.016). PSEQ scores were only significant in terms of planned pregnancy (p = 0.000), but did not show a significant difference in terms of receiving preconception care/counseling (p = 0.857), (Table 5).

**Table 5:** Comparison of PKAS and PSEQ scores by pregnancy planning and preconception care status (n = 388) – Uşak, TR, Türkiye, 2024.

Variable	Category	n (%)	PKAS Mean ± SD	U	p	PSEQ Mean ± SD	U	p
Pregnancy planning	Planned	284 (73.2)	195.79 ± 15.61	12189.500	0.008*	129.71 ± 25.24	10425.000	0.000*
	Unplanned	104 (26.8)	190.34 ± 19.07			143.69 ± 29.13		
Preconception Care Status	Yes	188 (48.5)	196.51 ± 15.40	16148.000	0.016*	133.47 ± 28.71	18601.500	0.857
	No	200 (51.5)	192.28 ± 17.74			133.45 ± 25.40		

Note: PKAS: Preconception Knowledge and Attitude Scale; PSEQ: Prenatal Self-Evaluation Questionnaire; SD: Standard deviation. U: Mann-Whitney U test test were used.

*p < 0.05 was considered statistically significant.

Women with planned pregnancies were significantly more likely to receive preconception care/counseling compared to those with unplanned pregnancies (χ^2^ = 6.826; p = 0.009) (Table 6).

**Table 6:** Association between preconception care/counseling and pregnancy planning – Uşak, TR, Türkiye, 2024.

Preconception care/counseling status	Planned pregnancy n (%)	Unplanned pregnancy n (%)	χ^2^	p
Yes	149 (79.3)	39 (20.7)	6.826	0.009*
No	135 (67.5)	65 (32.5)		

Chi-square: χ^2^ p < 0.05 was considered statistically significant.

### Associations with Sociodemographic and Obstetric Variables

Sociodemographic and obstetric variables were evaluated as potential confounding factors, and their associations with scale scores are presented in Supplementary Tables S1–S2. Age, educational level, occupation, social security status, smoking status, planned pregnancy, and history of stillbirth were found to be significantly associated with PSEQ and PKAS scores and some subscales (p < 0.05). In particular, it was determined that as educational level increased, PSEQ scores decreased (better adaptation to pregnancy) and PKAS scores increased (higher knowledge and more positive attitudes). The relationships observed for other variables were limited and varied depending on the subdimensions. In contrast, no significant relationship was found between either scale and chronic illness, consanguineous marriage, or previous pregnancy/birth complications (p > 0.05).

## DISCUSSION

The results supported Hypothesis 2 by showing significant correlations between preconception knowledge and attitudes and adaptation to pregnancy. Higher levels of knowledge and more positive attitudes were associated with better psychological, emotional, and social adaptation during pregnancy. In contrast, no significant direct relationship was found between receiving preconception care/counseling and adaptation to pregnancy; therefore, Hypothesis 1 was not supported. However, preconception care/counseling was significantly associated with higher knowledge and attitude scores; this finding suggests that the effect of preconception care/counseling on adaptation to pregnancy may not be direct, but rather may be mediated through knowledge and attitudes.

In our study, approximately half of pregnant women (48.5%) received care/counseling during the preconceptional period. This finding indicates that, despite the widespread availability of these services, there are still significant gaps in their uptake. The literature also supports the view that preconceptional care varies between countries. For example, in Ethiopia, only 10% of women received preconceptional care^(17)^, whereas in Malawi, it was found that 57.7% of pregnant women were well-informed on this matter^(18)^. In another study, the service utilisation rate was only 1.5%^(19)^. A meta-analysis evaluating the rates of preconception care uptake among women in Africa found an average rate of 18.72%, with marked differences observed between countries^(20)^. Similarly, in Ghana, this rate ranged from 14.3% to 25.0% among postpartum women^(21)^, whilst in Addis Ababa, it was reported to be 40% among women attending private maternal and child health hospitals^(22)^. These data indicate that differences in service utilisation are linked to access to healthcare, public awareness and health systems.

In our study, when examining the topics covered during preconception care/counselling, folic acid and vitamin intake (30.2%) and vaccination (21.6%) were the most frequently addressed topics. The rates of counselling received on smoking cessation (3.1%), alcohol cessation (1.5%) and genetic disorders (5.2%) were found to be low. This finding indicates that preconception care tends to focus primarily on biomedical aspects, while lifestyle modifications and genetic risks are addressed to a more limited extent.

Ayele et al.^(23)^ reported that 60.7% of women received counselling on screening for sexually transmitted infections/HIV and 38.4% on folic acid and iron supplements. These findings indicate that the content of the counselling provided is heterogeneous and that services should be delivered using a more holistic approach.

The present study found a significant negative correlation between the PKAS and the PSEQ. When the scoring systems of the scales are taken into account, this relationship actually indicates a positive situation; low scores on the PSEQ indicate high adaptation, whilst high scores on the PKAS indicate positive knowledge and attitude. Consequently, as the level of knowledge and attitude increases, so does adaptation to pregnancy. The literature also supports the notion that adaptation to pregnancy is associated with psychosocial factors and that antenatal education can enhance adaptation^(24)^. Teskereci et al.^(25)^, however, reported significant associations between adaptation to pregnancy and prenatal attachment and maternal self-confidence.

Specifically regarding the PSEQ subscales, negative correlations in the ‘Preparation for Labour’, ‘Fear of Helplessness and Loss of Control in Labour’, ‘Identification of a Motherhood Role’ and ‘Relationship with Her Husband/Partner’ dimensions indicate that preconception knowledge and attitude levels enhance not only general adaptation to pregnancy but also psychosocial adaptation. The literature reports that preconception care and childbirth preparation programmes reduce fear of childbirth and anxiety, and support psychosocial adaptation^(25,26)^.

An analysis of the PKAS sub-dimensions reveals that the low care/counselling rates in the ‘Attitudes Toward of Previous Life Conditions Before Having a Child’ and ‘Attitudes Towards of Preconceptional Risk Factors’ dimensions indicate that women are not sufficiently informed about daily life and potential risks. Similarly, the low rates of implementation in the “Attitudes Towards of Preconceptional Health Behaviors” and “Behaviors that should be Avoided in the Preconceptional Period” sub-dimensions reveal that healthy lifestyle changes are not sufficiently supported. The literature also emphasises that preconception care is predominantly focused on biomedical measures, whilst psychosocial and behavioral dimensions are not adequately addressed^(18,19,20,26)^. Therefore, programmes that encompass biomedical, behavioral and psychosocial dimensions offer a holistic approach that will improve maternal and foetal health by enabling women to make informed decisions.

Our study demonstrates that both planned pregnancy and preconception care/counseling increase women’s levels of knowledge and attitudes regarding pregnancy. However, it was observed that planned pregnancy has a significant association with pregnancy adjustment. Furthermore, significant correlations were found between preconception knowledge and attitude levels and pregnancy adjustment. While significant differences were found in PKAS scores based on whether women had a planned pregnancy or received preconceptional care/counseling, significant differences in PSEQ scores were observed only for planned pregnancy; no significant difference was found for those who received preconception care/counseling. These findings suggest that preconception care/counseling is effective in improving knowledge and attitudes but does not show a direct, significant relationship with pregnancy adaptation. Consequently, it is suggested that planned pregnancy and levels of knowledge and attitudes may be more decisive factors in pregnancy adaptation, while counseling may play an indirect role.

Sociodemographic and obstetric variables were considered as potential confounding factors. Analyses showed that some sociodemographic factors, such as age, education, occupation, social security, smoking, and planned pregnancy, were significantly associated with PSEQ scores and subscales; PKAS scores were similarly affected by sociodemographic factors, but preconceptional care/counseling was not significantly associated with PKAS. Chronic disease, consanguineous marriage, and previous pregnancy/childbirth complications in general were not associated with either scale, although limited effects were observed in some subscales. These findings suggest that sociodemographic factors may influence the relationships between adaptation to pregnancy and preconceptional knowledge/attitudes, while a history of obstetric risk is not a determinant in the overall scale.

In conclusion, levels of preconception knowledge and attitudes play a critical role in supporting both biomedical and psychosocial well-being. Therefore, care services must be delivered through a holistic approach that encompasses behavioral and psychosocial dimensions, rather than being limited to biomedical interventions alone.

### Bias/Limitations

This study has several limitations. First, due to its cross-sectional design, causal relationships could not be established. Second, data were collected from pregnant women presenting to a single hospital in the Central Aegean Region in Türkiye; this may limit the generalizability of the findings. Furthermore, potential sources of bias should be considered. Participants were drawn from a hospital setting; this may have led to selection bias, as the sample may not fully represent the general population of pregnant women. Self-report bias, particularly for variables such as smoking, alcohol use, and attitudes, may have influenced the results. Recall bias is also possible, as participants were asked to report their preconceptional experiences. Although potential confounding variables were considered in the analyses, residual confounding effects cannot be completely ruled out.

## CONCLUSION

This study revealed a statistically significant relationship between pregnant women’s knowledge and attitudes regarding preconception care and their adaptation to pregnancy; it is believed that increased knowledge and attitudes positively impact adaptation to pregnancy. The fact that only approximately half of the participants received care/counseling during this period suggests a low level of awareness and knowledge regarding preconception care.

Counseling services appear to focus primarily on biomedical issues, while areas focused on psychosocial support remain open to improvement. The findings indicate that information and support provided during the preconception period enhances women’s adaptation to pregnancy, and that psychological and social preparation is at least as important as physical preparation during this process.

The significant relationships observed between preconception attitudes and psychosocial dimensions, such as, Fear of Helplessness and Loss of Control in Labour, Identification of a Motherhood Role, and Relationship with Her Husband/Partner, indicate that this period should be addressed with a holistic approach, not just a medical one. Quality healthcare services and counseling provided during the preconception period can positively impact the health of both mother and baby. Therefore, it is crucial to develop health policies to increase the accessibility and coverage of preconception care. Effective care and counseling during the preconception period can prevent maternal, fetal, and neonatal complications and reduce maternal, fetal, and neonatal mortality. This preventive approach can also reduce the need for prenatal and postnatal health services, alleviating the financial burden on the healthcare system. Therefore, expanding and increasing the accessibility of preconception care will yield health benefits at both the individual and societal levels.

## Data Availability

The dataset created and/or analyzed during the current study is available from the corresponding author upon reasonable request.

## SUPPLEMENTARY MATERIAL

The following online material is available for this article.

Table S1 – Associations Between Sociodemographic and Obstetric Variables and PKAS Scores and Subscales.

Table S2 – Associations Between Sociodemographic and Obstetric Variables and PSEQ Scores and Subscales.
