# Supplementary material for: The relationship between pregnant women’s attitudes toward preconception care and pregnancy adaptation: a cross-sectional descriptive study
Source: Rev Esc Enferm USP. 2026 Jul 17;60:e20250468. doi: 10.1590/1980-220X-REEUSP-2025-0468en (PMC13379226; doi:10.1590/1980-220X-REEUSP-2025-0468en)
Supplement: Supplementary file 1 [file 1980-220X-reeusp-60-e20250468-suppl1.pdf]

Rev Esc Enferm USP

https://doi.org/10.1590/1980-220X-REEUSP-2025-0468en

Supplementary Material to "The relationship between pregnant women's attitudes toward preconception care and pregnancy adaptation: a cross-sectional descriptive study"

Table S1 - Associations Between Sociodemographic and Obstetric Variables and PKAS Scores and Subscales.

**Variables**		Preconception Care Knowledge and Attitude Scale			Attitudes Toward Protecting and Improving Preconception Health			Attitudes Toward Family Planning			Attitudes Regarding Living Conditions Before Having Children			Attitudes Toward Preconception Risk Factors			Attitudes Toward Preconception Health Behaviors			Behaviors to Avoid During the Preconception Period			Sensitivity to Preconception Health		
		**X̄**	**SD**	M	**X̄**	**SD**	M	**X̄**	**SD**	M	**X̄**	**SD**	M	**X̄**	**SD**	M	**X̄**	**SD**	M	**X̄**	**SD**	M	**X̄**	**SD**	M
**Age**	18-23[1]	187,96	18,66	191,00	45,03	5,07	46,00	30,92	3,62	31,00	20,82	3,03	21,00	34,93	4,18	36,00	20,70	3,58	21,00	21,70	3,46	23,00	13,86	1,95	15,00
	24–29[2]	196,48	15,24	200,00	47,00	4,31	48,00	31,92	3,77	33,00	21,60	3,06	22,00	36,76	4,11	38,00	21,78	3,48	23,00	23,09	2,53	24,00	14,33	1,33	15,00
	30–35[3]	195,82	16,76	202,00	47,11	3,52	48,00	31,50	3,46	32,00	21,68	3,31	23,00	36,55	3,82	38,00	21,60	3,51	23,00	23,10	2,82	25,00	14,27	1,29	15,00
	36-45[4]	191,55	17,33	197,00	46,61	3,91	48,00	31,03	3,40	31,00	20,79	3,44	22,00	35,33	4,08	36,00	21,18	5,68	21,00	22,73	2,36	23,00	13,88	1,67	15,00
H-test		13,384			8,997			7,541			6,591			15,696			7,742			11,353			4,398		
p		0,004*			0,029*			0,057			0,086			0,001*			0,052			0,010*			0,222		
Post Hoc		2,3>1			2>1			-			-			2,3>1			-			2,3>1			-		
**Educational status**	Primary school graduate or below[1]	184,34	20,06	186,50	44,34	6,18	46,00	29,90	3,99	31,00	20,64	3,43	21,00	34,18	4,85	35,00	20,24	4,22	21,50	21,54	3,78	23,00	13,50	2,26	15,00
	Secondary school graduate	191,40	18,41	197,50	45,95	4,54	48,00	31,21	4,03	32,00	21,05	3,36	21,00	35,49	4,70	37,00	21,21	4,23	22,00	22,49	3,00	23,00	14,01	1,58	15,00

Variables		Preconception Care Knowledge and Attitude Scale			Attitudes Toward Protecting and Improving Preconception Health			Attitudes Toward Family Planning			Attitudes Regarding Living Conditions Before Having Children			Attitudes Toward Preconception Risk Factors			Attitudes Toward Preconception Health Behaviors			Behaviors to Avoid During the Preconception Period			Sensitivity to Preconception Health		
		$\bar{X}$	SD	M	$\bar{X}$	SD	M	$\bar{X}$	SD	M	$\bar{X}$	SD	M	$\bar{X}$	SD	M	$\bar{X}$	SD	M	$\bar{X}$	SD	M	$\bar{X}$	SD	M
	College [2]																								
	College/ University graduate[3]	198,51	12,43	202,00	47,64	3,07	49,00	32,09	3,09	33,00	21,84	2,94	22,00	37,22	2,96	38,00	21,87	3,08	22,00	23,36	2,17	24,50	14,49	1,01	15,00
	Master's / Doctorate degree [4]	205,33	9,76	208,00	48,83	1,58	50,00	33,72	1,71	34,50	22,22	2,16	22,50	38,83	1,62	40,00	23,50	2,66	25,00	23,61	2,55	25,00	14,61	1,14	15,00
H-test		36,245			28,349			20,504			7,246			28,123			13,914			14,163			15,570		
p		0,000*			0,000*			0,000*			0,064			0,000*			0,003*			0,003*			0,001*		
Post Hoc		3,4>1,2			3,4>1,2			3,4>1 3>2			-			3,4>1,2			4>1,2			3,4>1			3>1		
**Consanguineous marriage status**	Yes	190,93	15,27	197,00	46,56	2,89	47,00	30,96	3,52	31,00	21,07	3,09	21,00	33,93	3,65	34,00	21,56	3,36	22,00	22,70	3,85	24,00	14,15	1,54	15,00
	No	194,58	16,85	200,00	46,64	4,38	48,00	31,60	3,65	33,00	21,43	3,17	22,00	36,42	4,08	38,00	21,48	3,77	22,00	22,81	2,74	24,00	14,19	1,49	15,00
U-test		3896,000			4262,500			4272,000			4491,000			2772,500			4867,000			4694,000			4873,000		
p		0,082			0,267			0,277			0,492			0,000*			0,991			0,737			0,999		
**Occupation**	Homemaker[1]	191,07	18,07	197,50	45,79	4,89	48,00	31,27	3,61	32,00	20,99	3,31	22,00	35,48	4,47	37,00	20,99	4,16	22,00	22,55	2,95	24,00	14,00	1,49	15,00
	Civil servant[2]	200,05	13,24	205,00	47,86	3,11	49,00	32,12	3,83	33,00	22,19	2,75	23,00	37,63	3,03	39,00	22,51	2,73	24,00	23,17	2,56	25,00	14,57	1,09	15,00
	Worker[3]	197,28	14,29	201,00	47,74	2,98	49,00	32,07	3,19	33,00	21,13	2,95	21,00	36,96	3,75	38,50	21,72	3,16	22,00	23,26	2,27	24,50	14,41	1,44	15,00
	Self emloyed[4]	196,72	13,82	202,00	47,64	2,23	48,00	31,00	3,84	32,00	22,76	2,88	24,00	36,72	3,05	38,00	21,72	3,37	23,00	22,88	3,37	25,00	14,00	2,38	15,00
H-test		21,349			19,866			6,187			15,164			22,209			10,919			4,326			14,113		
p		0,000*			0,000*			0,103			0,002*			0,000*			0,012*			0,228			0,003*		
Post Hoc		2>1			2>1			-			2,3>1			2>1			2>1			-			2>1		
	Yes	188,67	18,14	195,00	45,59	4,33	46,00	30,46	3,86	31,00	21,03	3,29	21,00	34,62	5,62	36,00	20,70	5,28	22,00	22,15	3,27	23,00	14,11	1,47	15,00

Variables		Preconception Care Knowledge and Attitude Scale			Attitudes Toward Protecting and Improving Preconception Health			Attitudes Toward Family Planning			Attitudes Regarding Living Conditions Before Having Children			Attitudes Toward Preconception Risk Factors			Attitudes Toward Preconception Health Behaviors			Behaviors to Avoid During the Preconception Period			Sensitivity to Preconception Health		
		**$\bar{X}$**	**SD**	M	**$\bar{X}$**	**SD**	M	**$\bar{X}$**	**SD**	M	**$\bar{X}$**	**SD**	M	**$\bar{X}$**	**SD**	M	**$\bar{X}$**	**SD**	M	**$\bar{X}$**	**SD**	M	**$\bar{X}$**	**SD**	M
**Social security status**	No	195,34	16,27	201,00	46,84	4,14	48,00	31,67	3,61	33,00	21,44	3,17	22,00	36,57	3,69	38,00	21,67	3,36	22,00	22,93	2,73	24,00	14,21	1,49	15,00
U-test		9,303			5,826			9,837			1,471			6,609			4,166			3,984			0,106		
p		0,010*			0,054			0,007*			0,479			0,037*			0,125			0,136			0,948		
**Chronic disease status**	Yes	194,35	17,26	199,00	46,84	3,96	48,00	31,35	3,76	32,00	20,87	3,55	22,00	36,19	4,50	38,00	21,77	5,73	22,00	23,03	2,36	24,00	14,29	1,35	15,00
	No	194,32	16,74	199,00	46,62	4,32	48,00	31,57	3,63	33,00	21,46	3,13	22,00	36,25	4,06	38,00	21,46	3,53	22,00	22,79	2,86	24,00	14,18	1,50	15,00
U-test		5510,000			5495,500			5396,500			5039,500			5279,500			5389,000			5398,500			5389,500		
p		0,969			0,948			0,816			0,405			0,667			0,806			0,812			0,769		
**History of still birth**	Yes[1]	200,88	11,62	204,00	48,27	1,99	49,00	32,23	3,29	33,00	22,46	2,37	23,00	37,19	3,62	38,50	22,19	3,30	23,00	23,65	3,31	25,00	14,88	0,43	15,00
	No[2]	191,87	17,79	199,00	46,10	4,72	48,00	31,23	3,91	32,00	20,86	3,49	21,00	35,93	4,02	38,00	21,16	4,11	22,00	22,71	2,92	24,00	13,89	1,79	15,00
	Nullipara[3]	195,55	16,11	199,00	46,87	4,06	48,00	31,74	3,43	33,00	21,74	2,89	22,00	36,40	4,21	38,00	21,67	3,45	23,00	22,77	2,67	24,00	14,35	1,22	15,00
H-test		8,311			5,489			2,441			7,464			4,566			4,135			5,376			13,583		
p		0,016*			0,064			0,295			0,024*			0,102			0,126			0,068			0,001*		
Post Hoc		1>2			-			-			1>2			-			-			-			1,3>2		
**Presence of complications threatening the previous pregnancy**	Yes	191,55	18,18	196,00	45,95	4,27	47,50	31,03	4,80	32,00	20,88	2,95	21,00	35,78	3,88	37,50	21,05	3,31	21,00	22,85	3,50	25,00	14,03	1,59	15,00
	No	193,49	17,16	200,00	46,51	4,58	48,00	31,45	3,56	33,00	21,13	3,51	22,00	36,18	4,02	38,00	21,36	4,19	22,00	22,84	2,84	24,00	14,02	1,75	15,00
	Primigravida	195,55	16,11	199,00	46,87	4,06	48,00	31,74	3,43	33,00	21,74	2,89	22,00	36,40	4,21	38,00	21,67	3,45	23,00	22,77	2,67	24,00	14,35	1,22	15,00
H-test		2,570			2,659			,687			3,739			2,670			2,053			0,900			2,866		
p		0,277			0,265			0,709			0,154			0,263			0,358			0,638			0,239		

**Variables**		Preconception Care Knowledge and Attitude Scale			Attitudes Toward Protecting and Improving Preconception Health			Attitudes Toward Family Planning			Attitudes Regarding Living Conditions Before Having Children			Attitudes Toward Preconception Risk Factors			Attitudes Toward Preconception Health Behaviors			Behaviors to Avoid During the Preconception Period			Sensitivity to Preconception Health		
		$\bar{X}$	**SD**	M	$\bar{X}$	**SD**	M	$\bar{X}$	**SD**	M	$\bar{X}$	**SD**	M	$\bar{X}$	**SD**	M	$\bar{X}$	**SD**	M	$\bar{X}$	**SD**	M	$\bar{X}$	**SD**	M
**Presence of complications in previous childbirth**	Yes	194,17	16,50	199,00	46,83	3,63	48,00	31,89	3,46	33,00	20,74	3,54	21,00	36,69	3,79	38,00	20,77	3,39	21,00	23,23	2,44	24,00	14,03	1,72	15,00
	No	192,85	17,57	199,50	46,30	4,69	48,00	31,25	3,92	32,00	21,15	3,37	22,00	35,97	4,02	38,00	21,41	4,14	22,00	22,75	3,09	24,00	14,02	1,71	15,00
	Primigravida	195,55	16,11	199,00	46,87	4,06	48,00	31,74	3,43	33,00	21,74	2,89	22,00	36,40	4,21	38,00	21,67	3,45	23,00	22,77	2,67	24,00	14,35	1,22	15,00
H-test		2,047			1,848			1,404			3,349			2,347			2,964			0,535			2,790		
p		0,359			0,397			0,496			0,187			0,309			0,227			0,765			0,248		
**Planned status of the current pregnancy**	Yes	195,79	15,61	200,00	47,02	3,65	48,00	31,82	3,43	33,00	21,60	3,10	22,00	36,58	3,87	38,00	21,59	3,43	22,00	22,87	2,78	24,00	14,30	1,40	15,00
	No	190,34	19,07	196,00	45,57	5,56	48,00	30,83	4,08	31,00	20,89	3,29	21,00	35,34	4,54	36,00	21,19	4,49	22,00	22,63	2,95	24,00	13,89	1,69	15,00
U-test		12189,500			12488,000			12457,000			12882,500			12230,500			13646,500			14136,000			12991,000		
p		0,008*			0,017*			0,017*			0,052			0,009*			0,245			0,496			0,026*		
**Smoking status**	Currently smoking	186,59	19,89	190,00	42,56	5,27	43,00	30,80	4,54	31,00	21,15	3,21	22,00	35,71	4,11	36,00	20,76	3,28	21,00	21,78	3,55	23,00	13,83	1,79	15,00
	Never smoked	194,67	16,53	200,00	47,11	4,01	49,00	31,57	3,55	33,00	21,25	3,27	22,00	36,11	4,28	38,00	21,48	3,86	22,00	22,90	2,66	24,00	14,26	1,36	15,00
	Quit due to pregnancy	198,25	13,63	203,00	47,13	3,25	48,00	32,04	3,33	33,00	22,45	2,28	23,00	37,42	2,66	38,00	22,07	3,35	23,00	23,07	3,00	25,00	14,07	1,85	15,00
H-test		10,559			35,378			2,405			5,575			3,233			4,913			4,156			2,219		
p		0,005*			0,000*			0,300			0,062			0,199			0,086			0,125			0,330		
Post Hoc		2,3>1			2,3>1			-			-			-			-			-			-		
**Receipt of counseling and**	Yes	196,51	15,40	202,00	47,10	3,51	48,00	31,66	3,63	33,00	21,79	3,02	22,50	36,79	3,75	38,00	21,88	3,43	23,00	22,96	2,68	24,00	14,32	1,43	15,00
	No	192,28	17,74	198,00	46,20	4,88	48,00	31,45	3,66	32,50	21,05	3,27	21,00	35,75	4,34	37,00	21,11	3,98	21,00	22,67	2,95	24,00	14,06	1,54	15,00

**Variables**		Preconception Care Knowledge and Attitude Scale			Attitudes Toward Protecting and Improving Preconception Health			Attitudes Toward Family Planning			Attitudes Regarding Living Conditions Before Having Children			Attitudes Toward Preconception Risk Factors			Attitudes Toward Preconception Health Behaviors			Behaviors to Avoid During the Preconception Period			Sensitivity to Preconception Health		
		**$\bar{X}$**	**SD**	M	**$\bar{X}$**	**SD**	M	**$\bar{X}$**	**SD**	M	**$\bar{X}$**	**SD**	M	**$\bar{X}$**	**SD**	M	**$\bar{X}$**	**SD**	M	**$\bar{X}$**	**SD**	M	**$\bar{X}$**	**SD**	M
**care services for the current pregnancy during the preconception period**																									
U-test		16148,000			17115,500			17838,000			16291,500			16306,000			16023,000			17794,500			17044,500		
p		0,016*			0,119			0,376			0,022*			0,022*			0,011*			0,338			0,052		

H: Kruskal-Wallis H test; used for comparing more than two independent groups.U: Mann-Whitney U test; used for comparing two independent groups. $p < 0.05$ indicates statistical significance.
