# Supplementary material for: The relationship between pregnant women’s attitudes toward preconception care and pregnancy adaptation: a cross-sectional descriptive study
Source: Rev Esc Enferm USP. 2026 Jul 17;60:e20250468. doi: 10.1590/1980-220X-REEUSP-2025-0468en (PMC13379226; doi:10.1590/1980-220X-REEUSP-2025-0468en)
Supplement: Supplementary file 2 [file 1980-220X-reeusp-60-e20250468-suppl2.pdf]

Rev Esc Enferm USP
https://doi.org/10.1590/1980-220X-REEUSP-2025-0468en

Supplementary Material to "The relationship between pregnant women's attitudes toward preconception care and pregnancy adaptation: a cross-sectional descriptive study"

Table 2 - Associations Between Sociodemographic and Obstetric Variables and PSEQ Scores and Subscales.

**Variables**		Prenatal Self-Evaluation Scale			Thoughts about own and baby's health			Acceptance of pregnancy			Acceptance of the maternal role			Preparedness for childbirth			Fear of childbirth			The status of her relationship with her mother			The status of her relationship with her spouse		
		**$\bar{X}$**	**SD**	M	**$\bar{X}$**	**SD**	M	**$\bar{X}$**	**SD**	M	**$\bar{X}$**	**SD**	M	**$\bar{X}$**	**SD**	M	**$\bar{X}$**	**SD**	M	**$\bar{X}$**	**SD**	M	**$\bar{X}$**	**SD**	M
**Age**	18-23[1]	137,68	26,75	136,00	22,31	5,92	22,00	21,46	5,47	20,00	23,32	5,90	21,00	18,66	5,19	18,00	21,37	4,33	22,00	15,00	5,60	13,00	15,55	5,44	14,00
	24–29[2]	130,57	26,21	123,00	22,06	5,95	22,00	21,17	5,44	20,00	22,86	5,24	22,00	16,96	4,11	17,00	19,70	4,38	20,00	13,82	5,02	12,00	14,01	4,44	13,00
	30–35[3]	131,98	25,68	127,00	22,23	6,90	22,00	20,50	6,21	19,00	22,54	4,72	22,00	17,50	5,14	17,00	20,13	5,04	21,00	14,43	5,13	13,00	14,65	5,14	13,00
	36-45[4]	144,79	32,72	141,00	21,33	7,14	21,00	25,00	7,42	23,00	24,97	6,40	23,00	17,88	4,04	16,00	19,94	4,85	20,00	17,48	7,20	15,00	18,18	7,36	18,00
H-test		9,387			0,709			14,514			3,404			5,020			6,893			9,678			13,219		
p		0,025*			0,871			0,002*			0,333			0,170			0,075			0,022*			0,004*		
Post Hoc		1,3>2			-			4>2,3			-			-			-			4>2			4>2		
**Educational status**	Primary school graduate or below [1]	142,46	26,11	140,50	23,04	5,51	23,50	22,50	4,94	21,00	23,66	5,53	22,50	18,58	4,41	18,00	20,14	4,30	20,00	17,28	5,92	17,00	17,26	5,84	17,00
	Secondary school graduate [2]	136,58	28,10	128,00	22,53	6,48	22,50	21,96	5,98	20,00	23,29	5,48	22,00	17,68	4,75	17,00	20,99	4,74	21,00	14,66	5,60	12,00	15,47	5,58	14,00
	College/University graduate[3]	129,62	25,14	126,50	21,71	6,44	21,00	20,78	6,07	19,00	22,79	5,16	22,00	17,33	4,54	17,00	19,70	4,52	20,00	13,49	4,50	12,00	13,83	4,35	13,00
	Master's / Doctorate degree [4]	120,17	28,76	109,00	19,56	4,69	19,00	19,22	6,04	17,00	21,72	5,80	22,00	14,50	4,02	13,50	17,44	4,15	19,00	15,39	8,14	12,00	12,33	5,31	11,00
H-test		17,549			5,759			16,775			2,092			12,641			13,904			18,672			24,679		
p		0,001*			0,124			0,001*			0,553			0,005*			0,003*			0,000*			0,000*		
Post Hoc		1,2>4			-			1,2>4			-			1,2,3>4			2>3,4			1>2,4			1,2>4		

Variables		Prenatal Self-Evaluation Scale			Thoughts about own and baby's health			Acceptance of pregnancy			Acceptance of the maternal role			Preparedness for childbirth			Fear of childbirth			The status of her relationship with her mother			The status of her relationship with her spouse		
		$\bar{X}$	SD	M	$\bar{X}$	SD	M	$\bar{X}$	SD	M	$\bar{X}$	SD	M	$\bar{X}$	SD	M	$\bar{X}$	SD	M	$\bar{X}$	SD	M	$\bar{X}$	SD	M
		1>3						1>3															1>3		
**Consanguineous marriage status**	Yes	134,15	25,67	134,00	22,96	6,81	24,00	20,59	5,12	19,00	22,78	4,52	22,00	17,33	3,87	17,00	20,63	4,61	22,00	14,67	5,17	13,00	15,19	4,43	14,00
	No	133,40	27,15	127,00	22,02	6,26	22,00	21,43	5,99	20,00	23,06	5,42	22,00	17,50	4,69	17,00	20,10	4,63	20,00	14,50	5,47	12,00	14,79	5,29	13,00
U-test		4609,500			4330,000			4418,000			4831,000			4859,000			4324,500			4758,000			4433,000		
p		0,639			0,333			0,416			0,940			0,979			0,328			0,835			0,429		
**Occupation**	Homemaker[1]	135,82	26,01	131,50	22,46	6,10	22,00	21,38	5,32	20,00	23,27	5,18	22,00	17,70	4,22	17,00	20,63	4,50	21,00	14,89	5,52	13,00	15,50	5,32	14,00
	Civil servant[2]	126,82	25,85	122,00	21,08	6,12	21,00	20,76	6,77	19,00	22,18	4,87	21,00	16,61	4,04	16,00	19,02	4,43	19,00	13,83	4,99	12,00	13,33	4,23	12,00
	Worker[3]	131,11	31,11	121,50	22,20	7,11	22,00	21,52	6,20	20,00	22,63	6,29	22,00	17,61	5,68	17,00	19,91	5,50	19,00	13,37	4,76	12,00	13,87	4,30	12,00
	Self emloyed[4]	141,32	28,47	129,00	22,36	7,07	24,00	23,32	7,22	20,00	24,96	6,45	24,00	18,72	7,21	18,00	20,24	4,08	20,00	15,76	7,05	13,00	15,96	7,63	14,00
H-test		11,103			3,819			4,228			6,295			3,968			9,694			5,668			14,454		
p		0,011*			0,282			0,238			0,098			0,265			0,021*			0,129			0,002*		
Post Hoc		1>2			-			-			-			-			1>2			-			1>2		
**Social security status**	Yes	145,54	29,47	140,00	24,52	6,49	25,00	23,13	6,08	22,00	25,16	5,06	26,00	18,36	4,23	18,00	21,61	4,26	22,00	15,48	5,64	13,00	17,28	6,99	16,00
	No	130,98	26,42	125,00	21,49	6,18	21,00	21,14	5,96	20,00	22,64	5,33	22,00	17,28	4,75	17,00	19,71	4,63	20,00	14,35	5,49	12,00	14,37	4,79	13,00
U-test		14,101			13,533			8,662			14,318			4,195			11,608			3,424			10,012		
p		0,001*			0,001*			0,013*			0,001*			0,123			0,003*			0,181			0,007*		
**Chronic disease status**	Yes	136,68	28,84	127,00	23,94	5,62	24,00	21,61	6,18	20,00	24,65	6,82	24,00	16,61	3,42	16,00	20,03	3,59	20,00	14,48	5,93	12,00	15,35	6,59	13,00
	No	133,18	26,88	128,00	21,93	6,33	22,00	21,35	5,92	20,00	22,90	5,20	22,00	17,57	4,72	17,00	20,15	4,70	21,00	14,51	5,41	12,00	14,77	5,10	13,00
U-test		5284,500			4430,500			5438,500			4635,000			4993,000			5291,000			5528,500			5350,500		
p		0,678			0,065			0,874			0,133			0,366			0,685			0,993			0,758		
**History of still birth**	Yes[1]	124,77	24,70	124,00	21,92	7,65	20,00	20,50	5,50	19,00	20,69	4,10	20,00	16,23	3,65	15,50	18,04	5,51	17,50	12,88	4,14	11,50	14,50	4,72	13,50
	No[2]	137,43	28,15	134,00	22,74	6,46	23,00	21,94	5,67	21,00	23,46	5,15	23,00	17,63	4,88	17,00	20,31	4,88	21,00	15,43	5,96	13,00	15,93	5,70	15,00
	Nullipara[3]	131,21	25,90	124,00	21,55	5,93	21,00	21,00	6,20	19,00	23,00	5,61	22,00	17,54	4,52	17,00	20,27	4,21	20,00	13,94	5,01	12,00	13,91	4,69	12,00
H-test		8,097			3,621			6,153			7,728			2,101			5,137			7,892			12,814		
p		0,017*			0,164			0,046*			0,021*			0,350			0,077			0,019*			0,002*		
Post Hoc		2>1,3			-			2>3			2>1			-			-			2>1,3			2>3		
**Presence of complications threatening the previous pregnancy**	Yes[1]	137,43	30,29	139,00	23,90	7,11	24,00	22,40	6,26	20,50	23,38	6,10	23,00	17,08	4,12	17,00	21,20	5,39	22,50	14,05	4,49	13,00	15,43	4,58	14,50
	No[2]	135,28	27,44	131,00	22,29	6,47	22,00	21,58	5,49	20,00	23,01	4,83	22,00	17,54	4,91	17,00	19,69	4,88	20,00	15,36	6,09	13,00	15,82	5,83	14,00
	Primigravida[3]	131,21	25,90	124,00	21,55	5,93	21,00	21,00	6,20	19,00	23,00	5,61	22,00	17,54	4,52	17,00	20,27	4,21	20,00	13,94	5,01	12,00	13,91	4,69	12,00
H-test		3,480			4,033			4,094			0,466			0,271			4,013			3,797			11,778		

Variables		Prenatal Self-Evaluation Scale			Thoughts about own and baby's health			Acceptance of pregnancy			Acceptance of the maternal role			Preparedness for childbirth			Fear of childbirth			The status of her relationship with her mother			The status of her relationship with her spouse		
		**$\bar{X}$**	**SD**	M	**$\bar{X}$**	**SD**	M	**$\bar{X}$**	**SD**	M	**$\bar{X}$**	**SD**	M	**$\bar{X}$**	**SD**	M	**$\bar{X}$**	**SD**	M	**$\bar{X}$**	**SD**	M	**$\bar{X}$**	**SD**	M
p		0,176			0,133			0,129			0,792			0,873			0,134			0,150			0,003*		
Post Hoc		-			-			-			-			-			-			-			2>3		
**Presence of complications in previous childbirth**	Yes[1]	139,23	29,06	138,00	24,74	7,43	26,00	21,83	5,34	22,00	23,34	5,36	23,00	17,83	4,03	17,00	21,51	5,44	21,00	13,80	4,41	12,00	16,17	5,50	15,00
	No[2]	134,95	27,78	131,00	22,16	6,35	22,00	21,73	5,74	20,00	23,03	5,06	22,00	17,36	4,91	16,00	19,66	4,86	20,00	15,37	6,05	13,00	15,64	5,62	14,00
	Primigravida[3]	131,21	25,90	124,00	21,55	5,93	21,00	21,00	6,20	19,00	23,00	5,61	22,00	17,54	4,52	17,00	20,27	4,21	20,00	13,94	5,01	12,00	13,91	4,69	12,00
H-test		4,211			6,450			4,037			0,663			0,931			4,028			5,625			12,209		
p		0,122			0,040*			0,133			0,718			0,628			0,133			0,060			0,002*		
Post Hoc		-			1>3			-			-			-			-			-			1,2>3		
**Planned status of the current pregnancy**	Yes	129,71	25,24	124,00	21,58	6,14	21,00	20,44	5,30	19,00	22,48	5,10	21,00	17,17	4,59	16,50	19,84	4,54	20,00	14,12	5,22	12,00	14,09	4,34	13,00
	No	143,69	29,13	138,50	23,48	6,53	24,00	23,92	6,79	22,00	24,59	5,76	24,00	18,37	4,64	18,00	20,95	4,77	22,00	15,59	5,92	13,00	16,80	6,75	15,00
U-test		10425,000			12151,500			9979,000			11429,000			12451,000			12468,000			12307,500			11601,500		
p		0,000*			0,007*			0,000*			0,001*			0,018*			0,018*			0,011*			0,001*		
**Smoking status**	Currently smoking[1]	145,98	28,22	143,00	25,05	7,75	27,00	23,34	6,33	22,00	25,00	5,48	24,00	19,12	4,48	19,00	22,46	5,09	22,00	14,76	5,54	12,00	16,24	6,16	14,00
	Never smoked[2]	132,61	26,65	127,00	21,62	6,09	21,00	21,21	5,93	20,00	22,90	5,32	22,00	17,41	4,53	17,00	19,91	4,48	20,00	14,66	5,45	13,00	14,89	5,28	13,00
	Quit due to pregnancy[3]	128,60	25,85	125,00	22,35	5,63	23,00	20,75	5,43	19,00	22,31	5,24	21,00	16,71	5,07	16,00	19,58	4,59	20,00	13,53	5,31	11,00	13,38	3,73	12,00
H-test		10,735			9,489			4,736			6,347			8,414			8,321			3,985			5,075		
p		0,005*			0,009			0,094			0,042			0,015			0,016			0,136			0,079		
Post Hoc		1>2,3			1>2			-			1>2,3			1>2			1>2,3			-			-		
**Receipt of counseling and care services for the current pregnancy during the preconception period**	Yes	133,47	28,71	128,00	21,88	6,52	22,00	21,55	6,42	20,00	23,32	5,76	22,00	17,61	4,93	17,00	19,87	4,62	20,00	14,55	5,80	12,00	14,68	5,42	13,00
	No	133,45	25,40	127,50	22,28	6,08	22,00	21,21	5,46	20,00	22,78	4,95	22,00	17,39	4,34	17,00	20,39	4,62	20,50	14,47	5,10	13,00	14,95	5,06	14,00
U-test		18601,500			18141,000			18766,500			18138,000			18436,000			18118,000			18301,500			17493,000		
p		0,857			0,550			0,976			0,548			0,741			0,536			0,648			0,232		

H: Kruskal-Wallis H test; used for comparing more than two independent groups.U: Mann-Whitney U test; used for comparing two independent groups. $p < 0.05$ indicates statistical significance.
