# Supplementary material for: The relationship between pregnant women’s attitudes toward preconception care and pregnancy adaptation: a cross-sectional descriptive study
Source: Rev Esc Enferm USP. 2026 Jul 17;60:e20250468. doi: 10.1590/1980-220X-REEUSP-2025-0468en (PMC13379226; doi:10.1590/1980-220X-REEUSP-2025-0468en)
Supplement: Translation

# Translation: La relación entre las actitudes de las mujeres embarazadas hacia la atención preconcepcional y la adaptación al embarazo: un estudio descriptivo transversal

**DOI:** 10.1590/1980-220X-reeusp-2025-0468es

**Authors:** Şeyma Çatalgöl, Zehra Üzel

**Affiliations:** 1 Uşak University Faculty of Health Sciences, Department of Nursing, Uşak, Türkiye.

**Keywords:** Atención Preconceptiva, Embarazo, Atención Prenatal

## Abstract

**Objetivo:**

El objetivo de este estudio fue examinar la relación entre los conocimientos y las actitudes de las mujeres embarazadas respecto a la atención preconcepcional y su adaptación al embarazo.

**Método:**

Este estudio descriptivo transversal se llevó a cabo entre el 26 de diciembre de 2023 y el 31 de julio de 2024, con 388 mujeres embarazadas de entre 18 y 45 años que acudieron al servicio de ginecología de un hospital de la región del Egeo Central de Turquía. Los datos se recopilaron mediante el Formulario de Información Introductoria, la Escala de Conocimientos y Actitudes Preconcepcionales (PKAS) y el Cuestionario de Autoevaluación Prenatal (PSEQ). Se utilizaron estadísticas descriptivas, coeficientes alfa de Cronbach y análisis de correlación de Pearson, con un nivel de significación establecido en p < 0,05.

**Resultados:**

Casi la mitad de las participantes (48,5%) recibió asesoramiento preconcepcional, principalmente sobre el ácido fólico y la vacunación. Se encontraron correlaciones negativas significativas entre las dos escalas y sus subescalas (p < 0,05). Dado que las puntuaciones más bajas en la escala PSEQ indican un mayor nivel de adaptación al embarazo, estas relaciones apuntan a una correlación positiva.

**Conclusión:**

Los resultados indican que mayores niveles de conocimientos y actitudes previos a la concepción facilitan la adaptación al embarazo y ponen de relieve la importancia de la atención preconcepcional para un embarazo y un parto saludables.

## INTRODUCCIÓN

Los servicios de atención y asesoramiento preconcepcional son un servicio de salud preventiva crucial que abarca intervenciones integrales para optimizar la salud antes del embarazo, con el objetivo de mejorar las tasas de embarazo, los resultados del embarazo, la salud infantil y la salud de las generaciones futuras^(1)^.

La atención y el asesoramiento preconcepcionales son un enfoque de prevención primaria que tiene como objetivo identificar los posibles riesgos para la salud de las mujeres y las parejas en edad reproductiva antes de la concepción, así como proporcionar una serie de intervenciones educativas y de manejo para abordar estos riesgos. Los riesgos incluyen factores biológicos, ambientales y conductuales. En este contexto, destacan los trastornos genéticos, la exposición a teratógenos, los hábitos de vida (tabaquismo, consumo de sustancias, dieta) y la deficiencia de ácido fólico^(2)^.

La atención y el asesoramiento preconcepcionales son especialmente importantes para las pacientes con afecciones de alto riesgo, que pueden tener repercusiones significativas a principios del primer trimestre, antes de que la mayoría de las mujeres comiencen la atención prenatal. Para las mujeres que deciden posponer el embarazo, la atención y el asesoramiento preconcepcionales ofrecen la oportunidad de proporcionar atención anticonceptiva^(3)^.

Algunos estudios han encontrado evidencia de que la exposición a teratógenos o a condiciones ambientales que pueden afectar la fertilidad en el período preconcepcional repercute en la salud física, mental, social y psicológica, y aumenta el riesgo de preeclampsia, diabetes gestacional y resultados fetales adversos^(4,5)^.

Una revisión sistemática encontró que intervenciones como retrasar la edad del primer embarazo y optimizar el intervalo entre embarazos, que se encuentran entre las prácticas de atención y asesoramiento preconcepcional, aumentaron el uso de anticonceptivos. Además, se ha informado que el uso de combinaciones de ácido fólico y hierro durante el período preconcepcional reduce la anemia, y que el uso de ácido fólico reduce la incidencia de defectos del tubo neural^(6)^.

La experiencia del embarazo a lo largo de la vida de una mujer influye en diversos aspectos de su estilo de vida y da lugar a cambios en su rol social. Este es un período de adaptación psicosocial para las mujeres, durante el cual pueden experimentar incertidumbres sobre el embarazo y la maternidad^(7)^. Las mujeres con problemas de salud física y mental durante el período preconcepcional corren un mayor riesgo durante el embarazo en comparación con las mujeres que no tienen tales problemas, y su adaptación al embarazo es más difícil^(8)^.

La adaptación al embarazo es el proceso de dar sentido a los cambios físicos y psicosociales de la madre y está influenciada por factores como el apoyo familiar, el estado de salud y las complicaciones del embarazo^(9)^. Los embarazos de alto riesgo y los problemas de salud pueden complicar la adaptación al embarazo y aumentar los niveles de estrés y ansiedad de las mujeres^(10)^. Por lo tanto, las parteras pueden facilitar la adaptación de las mujeres al embarazo y prepararlas mejor para la maternidad a través del apoyo que brindan a las mujeres embarazadas y de experiencias positivas del embarazo^(11)^.

Preparar a las mujeres para el embarazo desde el período preconcepcional, identificar y tratar las condiciones de riesgo existentes antes de la concepción, brindar servicios de salud preventivos y prepararlas psicológica y físicamente para el embarazo pueden facilitar la adaptación de las mujeres al embarazo y a la maternidad.

Si bien la atención y el asesoramiento preconcepcionales tienen un impacto significativo en la salud maternoinfantil, hay poca evidencia sobre la relación entre los conocimientos y las actitudes de las mujeres embarazadas turcas hacia la atención preconcepcional y su adaptación al embarazo. Esta brecha de conocimiento es particularmente importante para comprender la prevalencia y el impacto de los servicios de atención y asesoramiento preconcepcionales. La presente investigación examina la relación entre los conocimientos y las actitudes preconcepcionales y la adaptación al embarazo entre las mujeres embarazadas en Turquía. En este contexto, se han planteado dos hipótesis: la hipótesis 1 predice que las mujeres que recibieron atención y asesoramiento preconcepcional tendrían mayores niveles de adaptación al embarazo que aquellas que no lo recibieron. La hipótesis 2 predice que las mujeres con más conocimientos sobre la atención preconcepcional tendrían mayores niveles de adaptación al embarazo que aquellas con menos conocimientos.

## MÉTODO

### Diseño del Estudio

Este estudio es un estudio transversal y descriptivo. La población del estudio consistió en todas las mujeres embarazadas que acudieron a la Clínica Ambulatoria de Obstetricia y Ginecología del Hospital de Formación e Investigación de Uşak entre el 26 de diciembre de 2023 y el 31 de julio de 2024 y que cumplían con los criterios del estudio. El informe del estudio se realizó de acuerdo con las directrices STROBE^(12)^.

### Criterios de Inclusión

- Mujeres embarazadas de entre 18 y 45 años,
- Entre 6 y 42 semanas de gestación,
- Que aceptaron voluntariamente participar en el estudio durante las fechas especificadas.

### Criterios de Exclusión

- Deterioro cognitivo o afecciones de salud graves que impidieran una participación confiable,
- Embarazos complicados por afecciones médicas graves que requirieran intervención especializada,
- Complicaciones, eventos intercurrentes o patologías que existieran antes del embarazo o surgieran durante el mismo.

### Muestra del Estudio

El tamaño de la muestra se calculó utilizando el software OpenEpi y la fórmula de población conocida n = [DEFF × N × p × (1–p)]/[(d^2^/Z^2^_1−_α/_2_ × (N–1)) + p × (1–p)]. Según el Instituto Turco de Estadística (TÜİK, 2022), se estimó que la población femenina de entre 18 y 45 años en Türkiye era de aproximadamente 17,8 millones, con base en los datos de población por grupos de edad del Sistema de Registro de Población Basado en Direcciones al 31 de diciembre de 2022. El tamaño mínimo de muestra requerido se calculó en 385 participantes. Considerando una posible pérdida de datos del 10 %, el tamaño de muestra objetivo se estableció en 428 participantes. Para alcanzar este objetivo, se contactó a 449 mujeres embarazadas, de las cuales 428 aceptaron participar en el estudio. Tras excluir a 40 participantes con datos incompletos, el análisis final se realizó con 388 participantes.

### Herramientas de Recopilación de Datos

A las mujeres embarazadas que cumplían los criterios del estudio y aceptaron participar tras ser informadas sobre el mismo se les entregó un formulario de información introductoria, y se les administró la Escala de Conocimientos y Actitudes Preconcepcionales (Preconception Knowledge and Attitude Scale – PKAS) junto con el Cuestionario de Autoevaluación Prenatal (Prenatal Self-Evaluation Questionnaire – PSEQ). Los datos fueron recopilados por los investigadores mediante un método de recopilación de datos presencial.

### Formulario de Información Descriptiva

Este formulario fue elaborado por los investigadores para determinar las características sociodemográficas y obstétricas de las mujeres embarazadas. Las características sociodemográficas incluían la edad, el nivel educativo, el estado civil, el matrimonio consanguíneo, la ocupación, la seguridad social, las enfermedades crónicas, el tabaquismo y el consumo de alcohol. La sección de características obstétricas constaba de 23 preguntas para evaluar el número de embarazos, los antecedentes obstétricos y la recepción de atención preconcepcional.

### Escala de Conocimientos y Actitudes Preconcepcionales (PKAS)

La escala, desarrollada por Gokdemir y Eryilmaz^(14)^, consta de 43 ítems y 7 subescalas. Las subescalas son:

1. Actitudes hacia la protección y la mejora de la salud preconcepcional
2. Actitudes hacia la planificación del embarazo
3. Actitudes hacia las condiciones de vida previas a tener un hijo
4. Actitudes hacia los factores de riesgo preconcepcionales
5. Actitudes hacia los comportamientos de salud preconcepcionales
6. Comportamientos que deben evitarse en el período preconcepcional
7. Estado de sensibilidad relacionado con la salud preconcepcional.

Los nombres de las subdimensiones se presentan tal como en la escala original. Consta de un total de 43 ítems, 42 positivos y uno negativo. El ítem 12 de la escala («Si mi matrimonio va mal, tendré un hijo para salvar mi matrimonio») se puntúa a la inversa. La escala es de tipo Likert de 5 puntos, y las puntuaciones más altas indican conocimientos y actitudes más positivos sobre la preconcepción. El coeficiente de confiabilidad (alfa de Cronbach) de la escala fue de 0,86, y el coeficiente de correlación test-retest fue de r = 0,978.

### Cuestionario de Autoevaluación Prenatal (PSEQ)

Este cuestionario es una escala de tipo Likert de 79 ítems y 4 puntos desarrollada por Lederman en 1979 para evaluar la adaptación de las mujeres a la maternidad durante el período prenatal. Beydağ y Mete evaluaron la validez y la fiabilidad de la escala en Turquía, y se determinó que el alfa de Cronbach era de 0,81. La escala tiene siete subdimensiones: Preocupación por el bienestar propio y del bebé, Aceptación del embarazo, Identificación con el rol de madre, Preparación para el parto, Miedo a la impotencia y a la pérdida de control durante el parto, Relación con la madre, Relación con el esposo/pareja. Cuarenta y siete de los ítems de la escala están codificados de forma inversa. Esta escala se evalúa en función de puntuaciones que van de 1 a 4 (4: Describe muy bien, 3: Describe algo, 2: Describe un poco, 1: No describe en absoluto). Para los ítems con codificación inversa, la puntuación se realiza en sentido contrario. La puntuación total de la escala puede oscilar entre un mínimo de 79 y un máximo de 316. Las puntuaciones más bajas indican un mayor nivel de adaptación al embarazo^(15,16)^. Por lo tanto, los valores negativos en los análisis de correlación indican una asociación positiva con una mayor adaptación al embarazo.

### Variables

#### Variables Independientes

Características sociodemográficas (edad, nivel educativo, estado civil, matrimonio consanguíneo, ocupación, seguridad social, enfermedades crónicas, tabaquismo, consumo de alcohol), antecedentes obstétricos (número de embarazos y partos, antecedentes de mortinatalidad y complicaciones experimentadas en embarazos o partos anteriores), factores relacionados con el embarazo (si el embarazo actual es planeado y si se recibió atención o asesoramiento preconcepcional) y los niveles de conocimiento y actitud preconcepcionales medidos por el PSEQ (puntuaciones totales y de subescalas) se incluyeron como variables independientes.

#### Variable Dependiente

La variable de resultado fue la adherencia al embarazo, evaluada mediante las puntuaciones totales y de las subescalas del PSEQ.

#### Variables de Confusión

Se consideraron como posibles variables de confusión la edad, el nivel educativo, el matrimonio consanguíneo, la ocupación, la situación en materia de seguridad social, la presencia de enfermedades crónicas, el tabaquismo, los antecedentes de mortinatalidad, la presencia de complicaciones que amenazaran el embarazo anterior, la presencia de complicaciones en partos anteriores, el carácter planificado del embarazo actual y la recepción de servicios de atención y asesoramiento para el embarazo actual durante el período preconcepcional.

### Análisis de Datos

Se utilizó el software Statistical Package for Social Sciences for Windows 25.0 (IBM Corp., Armonk, NY, EE. UU.) para analizar los datos. Las estadísticas descriptivas de los datos utilizados en el estudio se presentaron como media y desviación estándar para las variables continuas, y como mediana, mínimo, máximo y porcentaje (%). Además, para las variables en las que se aplicaron pruebas paramétricas y, especialmente, no paramétricas, las estadísticas descriptivas se presentaron en tablas como media-desviación estándar y mediana (mín.-máx.). Se adoptó un nivel de significación de p < 0,05 para determinar si existía una diferencia estadísticamente significativa entre los grupos. Al comparar dos grupos independientes, se utilizó la prueba t para muestras independientes cuando las variables continuas seguían una distribución normal, y la prueba U de Mann-Whitney cuando no se cumplía el supuesto de distribución normal. Se utilizó el análisis de varianza unidireccional (ANOVA) para comparar tres o más grupos independientes que seguían una distribución normal. Cuando no se cumplían los supuestos, se aplicó la prueba H de Kruskal-Wallis. Se utilizó la prueba de chi-cuadrado para comparar variables demográficas, variables categóricas y escalas y subescalas. Se utilizó el análisis de correlación de Pearson para determinar la relación entre las variables cuando se lograba una distribución normal, y el análisis de correlación de Spearman cuando no se lograba una distribución normal.

### Aspectos Éticos

Este estudio se llevó a cabo de conformidad con los principios de la Declaración de Helsinki. Se obtuvo la aprobación del Comité de Ética en Investigación Clínica No Intervencionista de la Universidad de Uşak (Fecha: 14 de diciembre de 2023; Decisión n.º: 267-267-34). Se obtuvo el permiso institucional para el estudio del Hospital de Investigación correspondiente (Fecha: 22 de noviembre de 2023; Número: E45786011-599-230168660).

## RESULTADOS

De las 449 mujeres embarazadas contactadas para participar en el estudio, 21 rechazaron participar y 428 fueron incluidas en el estudio. Tras la exclusión de 40 participantes con datos incompletos, la muestra analítica final quedó constituida por 388 participantes.

### Características Sociodemográficas de las Participantes

Las características sociodemográficas de las participantes se presentan en la Tabla 1. Se observó que el 46,6 % de las participantes tenían entre 24 y 29 años, el 44,8 % eran graduadas universitarias, el 99,2 % estaban casadas y el 7 % tenían matrimonios consanguíneos. Además, el 57,7 % eran amas de casa, el 84,3 % contaban con cobertura de seguridad social, el 8 % informaron tener una enfermedad crónica (estas incluían afecciones manejables que no requerían intervención especializada, por ejemplo, asma leve, hipotiroidismo controlado, diabetes controlada con dieta), el 10,6 % fumaban durante su embarazo actual y el 0,8 % consumían alcohol durante el embarazo (Tabla 1).

**Tabla 1:** Características sociodemográficas de las participantes – Uşak, TR, Turquía, 2024.

Variables	n	%
**Edad**		
18–23	71	18,3
24–29	181	46,6
30–35	103	26,5
36–45	33	8,5
**Nivel educativo**		
Primaria o inferior	50	12,9
Educación secundaria	146	37,6
Graduado universitario	174	44,8
Posgrado (máster/doctorado)	18	4,6
**Estado civil**		
Casado	385	99,2
Soltero	3	0,8
**Estado civil de matrimonio consanguíneo**		
Sí	27	7,0
No	361	93,0
**Ocupación**		
Ama de casa	224	57,7
Funcionaria	93	24,0
Obrero	46	11,9
Autónomo	25	6,4
**Situación en materia de seguridad social**		
Sí	327	84,3
No	61	15,7
*Estado de enfermedad crónica		
Sí	31	8,0
No	357	92,0
**Situación respecto al tabaquismo**		
Fumador actual	41	10,6
Nunca ha fumado	292	75,3
Dejó de fumar por embarazo	55	14,2
**Situación respecto al consumo de alcohol**		
Consumo actual	3	0,8
Nunca ha consumido	342	88,1
Dejó de fumar por embarazo	43	11,1
**Total**	**388**	**100,0**

*Las enfermedades crónicas incluían afecciones controlables que no requerían intervención especializada (p. ej., asma leve, hipotiroidismo controlado, diabetes controlada con dieta).

### Características Obstétricas y Estado de la Atención Preconcepcional de las Participantes

Las características obstétricas y el estado de la atención preconcepcional de las participantes se presentan en la Tabla 2. La mitad de las participantes (50 %) se encontraba en su primer embarazo (primigrávidas). Entre las 194 mujeres multíparas, el 12,4 % informó antecedentes de aborto espontáneo, el 8 % informó antecedentes de muerte fetal, el 10,3 % experimentó una complicación que supuso un riesgo para el embarazo y el 9 % informó haber experimentado complicaciones durante el parto. En general, el 73,2 % de las participantes afirmó que su embarazo actual era planeado (Tabla 2).

**Tabla 2:** Características obstétricas de las participantes y estado de la atención preconcepcional – Uşak, TR, Turquía, 2024.

Variables	n	%
**Número de embarazos**		
Primigrávidas	194	50
Multigrávida	194	50
**Antecedentes de aborto espontáneo**		
Sí	48	12,4
No	340	87,6
**Antecedentes de mortinatalidad**		
Sí	31	8
No	357	92
**Número de hijos vivos**		
Nullíparas	212	54,7
Primíparas	104	26,8
Multíparas	72	18,5
**Presencia de complicaciones que pusieron en peligro el embarazo anterior**		
Sí	40	10,3
No	154	39,7
Primigrávida	194	50
**Presencia de complicaciones en partos anteriores**		
Sí	35	9,0
No	159	41
Primigrávida	194	50
**Estado planificado del embarazo actual**		
Sí	284	73,2
No	104	26,8
**Recepción de servicios de atención o asesoramiento para el embarazo actual durante el periodo previo a la concepción**		
Sí	188	48,5
No	200	51,5
**Temas tratados durante el asesoramiento preconcepcional Dejar de fumar**		
Sí	12	3,1
No	376	96,9
**Dejar de beber**		
Sí	6	1,5
No	382	98,5
**Consumo de ácido fólico/vitaminas**		
Sí	117	30,2
No	271	69,8
**Estado de vacunación**		
Sí	84	21,6
No	304	78,4
**Tratamiento y gestión de enfermedades crónicas**		
Sí	25	6,4
No	363	93,6
**Alimentación adecuada y equilibrada**		
Sí	79	20,4
No	309	79,6
**Control del peso**		
Sí	64	16,5
No	324	83,5
**Enfermedades genéticas hereditarias**		
Sí	20	5,2
No	368	94,8
**Matrimonio consanguíneo**		
Sí	7	1,8
No	381	98,2
**Otros**		
Sí	2	0,5
No	386	99,5
**Total**	**388**	**100,0**

Se observó que el 48,5 % de las participantes recibió servicios de atención o asesoramiento durante el periodo previo a la concepción de su embarazo actual. Los temas más frecuentes fueron el consumo de ácido fólico o vitaminas (30,2 %), la vacunación (21,6 %), una nutrición adecuada y equilibrada (20,4 %) y el control del peso (16,5 %). Otros temas fueron el control de enfermedades crónicas (6,4 %), las enfermedades hereditarias (5,2 %), el abandono del tabaquismo (3,1 %), los matrimonios consanguíneos (1,8 %), el abandono del consumo de alcohol (1,5 %) y otros temas (0,5 %).

### Puntuaciones de las Escalas y Confiabilidad de los Participantes

Las estadísticas descriptivas y los coeficientes de fiabilidad de las escalas utilizadas en el estudio se presentan en la Tabla 3. La puntuación media de la PKAS fue de 194,32 ± 16,76 (α de Cronbach = 0,901), y la puntuación media del PSEQ fue de 133,46 ± 27,02 (α de Cronbach = 0,922). Se observó que la consistencia interna de ambas escalas, incluidas sus subdimensiones, era alta (Tabla 3).

**Tabla 3:** Estadísticas descriptivas de las puntuaciones de la PKAS y el PSEQ – Uşak, TR, Turquía, 2024.

Escalas y sus dimensiones	Mín	Máx	Media	DE	Alfa de Cronbach
**Escala de conocimientos y actitudes sobre la atención preconcepcional**	141,00	215,00	194,32	16,76	0,901
Actitudes hacia la salud preconcepcional y su mejora	14,00	50,00	46,63	4,29	0,746
Actitudes hacia la planificación del embarazo	10,00	35,00	31,55	3,64	0,749
Actitudes hacia las condiciones de vida anteriores antes de tener un hijo	9,00	25,00	21,41	3,17	0,812
Actitudes hacia los factores de riesgo preconcepcionales	15,00	40,00	36,25	4,09	0,729
Actitudes hacia los comportamientos de salud preconcepcionales	10,00	45,00	21,48	3,74	0,813
Comportamientos que deben evitarse durante el periodo preconcepcional	9,00	25,00	22,81	2,83	0,811
Relacionado con el estado de sensibilidad de la salud preconcepcional	4,00	15,00	14,19	1,49	0,799
**Cuestionario de autoevaluación prenatal**	83,00	230,00	133,46	27,02	0,922
Preocupación por el bienestar propio y del bebé	10,00	38,00	22,09	6,30	0,806
Aceptación del embarazo	14,00	62,00	21,37	5,94	0,797
Identificación del rol de la maternidad	15,00	44,00	23,04	5,36	0,823
Preparación para el parto	10,00	47,00	17,49	4,63	0,744
Miedo a la impotencia y a la pérdida de control durante el parto	10,00	34,00	20,14	4,62	0,873
Relación con su madre	9,00	37,00	14,51	5,44	0,870
Relación con su marido/pareja	10,00	40,00	14,82	5,23	0,828

Mín.: MínimoMáx.: MáximoSD: Desviación estándar.

### Correlaciones Entre las Puntuaciones del PSEQ y de la PKAS y sus Subscalas

La relación entre el PSEQ y sus subescalas y la PKAS y sus subescalas se presenta en la Tabla 4. Se identificó una correlación negativa estadísticamente significativa entre las puntuaciones totales del PSEQ y de la PKAS (r = −0,428, p < 0,001, moderada) (Tabla 4).

**Tabla 4:** Correlaciones entre las puntuaciones de la PKAS y el PSEQ y sus subescalas – Uşak, TR, Turquía, 2024.

Escalas y dimensiones		Escala de autoevaluación prenatal	Preocupación por el bienestar propio y del bebé	Aceptación del embarazo	Identificación del rol de madre	Preparación para el parto	Miedo a la impotencia y a la pérdida de control durante el parto	Relación con su madre	Relación con su marido/pareja
Escala de conocimientos y actitudes sobre la atención preconcepcional	r	-0,428**	-0,177**	-0,330**	-0,306**	-0,436**	-0,345**	-0,373**	-0,372**
	p	0,000	0,000	0,000	0,000	0,000	0,000	0,000	0,000
Actitudes hacia la salud preconcepcional	r	-0,376**	-0,177**	-0,257**	-0,272**	-0,321**	-0,296**	-0,303**	-0,365**
	p	0,000	0,000	0,000	0,000	0,000	0,000	0,000	0,000
**y la mejora**									
Actitudes hacia la planificación del embarazo	r	-0,337**	-0,155**	-0,273**	-0,258**	-0,268**	-0,230**	-0,294**	-0,345**
	p	0,000	0,002	0,000	0,000	0,000	0,000	0,000	0,000
Actitudes hacia las condiciones de vida anteriores	r	-0,242**	-0,060	-0,194**	-0,135**	-0,282**	-0,200**	-0,254**	-0,209**
	p	0,000	0,240	0,000	0,008	0,000	0,000	0,000	0,000
**antes de tener un hijo**									
Actitudes hacia los factores de riesgo preconcepcionales	r	-0,288**	-0,085	-0,201**	-0,161**	-0,305**	-0,234**	-0,280**	-0,281**
	p	0,000	0,094	0,000	0,001	0,000	0,000	0,000	0,000
Actitudes hacia los comportamientos de salud preconcepcionales	r	-0,313**	-0,065	-0,231**	-0,229**	-0,383**	-0,289**	-0,333**	-0,264**
	p	0,000	0,205	0,000	0,000	0,000	0,000	0,000	0,000
Comportamientos que deben evitarse en el periodo preconcepcional	r	-0,300**	-0,102*	-0,221**	-0,235**	-0,328**	-0,258**	-0,301**	-0,201**
	p	0,000	0,044	0,000	0,000	0,000	0,000	0,000	0,000
Relacionado con el estado de sensibilidad de la salud preconcepcional	r	-0,288**	-0,093	-0,201**	-0,185**	-0,321**	-0,198**	-0,279**	-0,313**
	p	0,000	0,067	0,000	0,000	0,000	0,000	0,000	0,000

*p < 0,05;

**p < 0,01; r: análisis de correlación de Pearson.

Además, se encontraron correlaciones negativas estadísticamente significativas entre la puntuación total del PSEQ y las subdimensiones de la PKAS. Estas correlaciones incluyeron: Actitudes hacia la protección y la mejora de la salud preconcepcional (r = −0,376, p < 0,001, moderada), Actitudes hacia la planificación del embarazo (r = −0,337, p < 0,001, moderada), Actitudes hacia las condiciones de vida previas a tener un hijo (r = −0,242, p < 0,001, débil), Actitudes hacia los factores de riesgo preconcepcionales (r = −0,288, p < 0,001, débil), Actitudes hacia los comportamientos de salud preconcepcionales (r = −0,313, p < 0,001, moderada), Comportamientos que deben evitarse en el periodo preconcepcional (r = −0,300, p < 0,001, moderada) y relacionadas con el estado de sensibilidad hacia la salud preconcepcional (r = −0,288, p < 0,001, débil).

Además, se observaron correlaciones negativas significativas entre la puntuación total de la PKAS y las subdimensiones del PSEQ. Estas oscilaron de débiles a moderadas, incluyendo: Preocupación por el bienestar propio y del bebé (r = −0,177, p < 0,001, débil), Aceptación del embarazo (r = −0,330, p < 0,001, moderada), Identificación del rol de maternidad (r = −0,306, p < 0,001, moderada), Preparación para el parto (r = −0,436, p < 0,001, moderada), Miedo a la impotencia y a la pérdida de control durante el parto (r = −0,345, p < 0,001, moderada), relación con su madre (r = −0,373, p < 0,001, moderada) y relación con su marido/pareja (r = −0,372, p < 0,001, moderada).

### Relación Entre la Planificación del Embarazo, la Atención/Asesoramiento Preconcepcional y las Puntuaciones de PKAS y PSEQ de las Participantes

La relación entre la planificación del embarazo, los servicios de atención/asesoramiento preconcepcional y las puntuaciones de las participantes en la PKAS y el PSEQ se presenta en la Tabla 5. Las puntuaciones de la PKAS mostraron diferencias significativas en cuanto al embarazo planificado (p = 0,008) y a la recepción de atención/asesoramiento preconcepcional (p = 0,016). Las puntuaciones del PSEQ solo fueron significativas en cuanto al embarazo planificado (p = 0,000), pero no mostraron una diferencia significativa en cuanto a la recepción de atención/asesoramiento preconcepcional (p = 0,857) (Tabla 5).

**Tabla 5:** Comparación de las puntuaciones de la PKAS y el PSEQ según la planificación del embarazo y el estado de la atención preconcepcional (n = 388) – Uşak, TR, Turquía, 2024.

Variable	Categoría	n (%)	PKAS Media ± DE	U	p	PSEQ Media ± DE	U	p
Planificación del embarazo	Planificado	284 (73,2)	195,79 ± 15,61	12189,500	0,008*	129,71 ± 25,24	10425,000	0,000*
	No planificado	104 (26,8)	190,34 ± 19,07			143,69 ± 29,13		
Estado de la atención preconcepcional	Sí	188 (48,5)	196,51 ± 15,40	16148,000	0,016*	133,47 ± 28,71	18601,500	0,857
	No	200 (51,5)	192,28 ± 17,74			133,45 ± 25,40		

Nota: PKAS: Escala de Conocimientos y Actitudes Preconcepcionales; PSEQ: Cuestionario de Autoevaluación Prenatal; DE: Desviación estándar. Se utilizó la prueba U de Mann-Whitney.

*Se consideró estadísticamente significativo un valor de p < 0,05.

Las mujeres con embarazos planificados eran significativamente más propensas a recibir atención o asesoramiento preconcepcional en comparación con aquellas con embarazos no planificados (χ^2^ = 6,826; p = 0,009) (Tabla 6).

**Tabla 6:** Asociación entre la atención y el asesoramiento preconcepcionales y la planificación del embarazo – Uşak, TR, Turquía, 2024.

Estado de la atención/asesoramiento preconcepcional	Embarazo planificado n (%)	Embarazo no planificado n (%)	χ^2^	p
Sí	149 (79,3)	39 (20,7)	6,826	0,009*
No	135 (67,5)	65 (32,5)		

Chi-cuadrado: χ^2^ p < 0,05 se consideró estadísticamente significativo.

### Asociaciones con Variables Sociodemográficas y Obstétricas

Se evaluaron las variables sociodemográficas y obstétricas como posibles factores de confusión, y sus asociaciones con las puntuaciones de las escalas se presentan en las Tablas complementarias S1 y S2. Se observó que la edad, el nivel educativo, la ocupación, la situación de seguridad social, el hábito de fumar, el embarazo planeado y los antecedentes de mortinatalidad se asociaban significativamente con las puntuaciones del PSEQ y la PKAS y con algunas subescalas (p < 0,05). En particular, se determinó que a medida que aumentaba el nivel educativo, las puntuaciones de la PSEQ disminuían (mejor adaptación al embarazo) y las puntuaciones de la PKAS aumentaban (mayor conocimiento y actitudes más positivas). Las relaciones observadas para otras variables fueron limitadas y variaron según las subdimensiones. Por el contrario, no se encontró ninguna relación significativa entre ninguna de las escalas y las enfermedades crónicas, el matrimonio consanguíneo o las complicaciones previas del embarazo o el parto (p > 0,05).

## DISCUSIÓN

Los resultados respaldaron la hipótesis 2 al mostrar correlaciones significativas entre los conocimientos y las actitudes previos a la concepción y la adaptación al embarazo. Un mayor nivel de conocimientos y actitudes más positivas se asociaron con una mejor adaptación psicológica, emocional y social durante el embarazo. Por el contrario, no se encontró una relación directa significativa entre recibir atención o asesoramiento preconcepcional y la adaptación al embarazo; por lo tanto, la hipótesis 1 no fue respaldada. Sin embargo, la atención o el asesoramiento preconcepcional se asoció significativamente con puntuaciones más altas en conocimientos y actitudes; este hallazgo sugiere que el efecto de la atención o el asesoramiento preconcepcional sobre la adaptación al embarazo puede no ser directo, sino que puede estar mediado por los conocimientos y las actitudes.

En nuestro estudio, aproximadamente la mitad de las mujeres embarazadas (48,5 %) recibió atención o asesoramiento durante el período preconcepcional. Este hallazgo indica que, a pesar de la amplia disponibilidad de estos servicios, todavía existen brechas significativas en su utilización. La literatura también respalda la opinión de que la atención preconcepcional varía entre países. Por ejemplo, en Etiopía, solo el 10 % de las mujeres recibió atención preconcepcional^(17)^, mientras que en Malaui se encontró que el 57,7 % de las mujeres embarazadas estaba bien informada sobre este tema^(18)^. En otro estudio, la tasa de utilización del servicio fue de solo el 1,5 %^(19)^. Un metaanálisis que evaluó las tasas de uso de la atención preconcepcional entre las mujeres en África encontró una tasa promedio del 18,72 %, con diferencias marcadas entre los países^(20)^. De manera similar, en Ghana, esta tasa osciló entre el 14,3 % y el 25,0 % entre las mujeres en el posparto^(21)^, mientras que en Addis Abeba se informó que era del 40 % entre las mujeres que acudían a hospitales privados de salud materno-infantil^(22)^. Estos datos indican que las diferencias en la utilización de los servicios están relacionadas con el acceso a la atención médica, la concienciación pública y los sistemas de salud.

En nuestro estudio, al examinar los temas tratados durante la atención o el asesoramiento preconcepcional, la ingesta de ácido fólico y vitaminas (30,2 %) y la vacunación (21,6 %) fueron los temas abordados con mayor frecuencia. Se observó que las tasas de asesoramiento recibido sobre el abandono del tabaquismo (3,1 %), el abandono del alcohol (1,5 %) y los trastornos genéticos (5,2 %) eran bajas. Este hallazgo indica que la atención preconcepcional tiende a centrarse principalmente en aspectos biomédicos, mientras que las modificaciones del estilo de vida y los riesgos genéticos se abordan en menor medida.

Ayele et al.^(23)^ informaron que el 60,7 % de las mujeres recibió asesoramiento sobre pruebas de detección de infecciones de transmisión sexual/VIH y el 38,4 % sobre suplementos de ácido fólico y hierro. Estos hallazgos indican que el contenido del asesoramiento brindado es heterogéneo y que los servicios deberían prestarse utilizando un enfoque más holístico.

El presente estudio encontró una correlación negativa significativa entre el PKAS y el PSEQ. Cuando se tienen en cuenta los sistemas de puntuación de las escalas, esta relación indica en realidad una situación positiva; las puntuaciones bajas en el PSEQ indican una alta adaptación, mientras que las puntuaciones altas en el PKAS indican conocimientos y actitudes positivos. En consecuencia, a medida que aumenta el nivel de conocimiento y actitud, también lo hace la adaptación al embarazo. La literatura también respalda la idea de que la adaptación al embarazo está asociada con factores psicosociales y que la educación prenatal puede mejorar la adaptación^(24)^. Teskereci et al.^(25)^, sin embargo, informaron de asociaciones significativas entre la adaptación al embarazo y el apego prenatal y la autoconfianza materna.

En lo que respecta específicamente a las subescalas del PSEQ, las correlaciones negativas en las dimensiones «Preparación para el parto», «Miedo a la impotencia y a la pérdida de control durante el parto», «Identificación con el rol de madre» y «Relación con su esposo/pareja» indican que los niveles de conocimiento y actitud previos a la concepción mejoran no solo la adaptación general al embarazo, sino también la adaptación psicosocial. La literatura indica que los programas de atención preconcepcional y preparación para el parto reducen el miedo al parto y la ansiedad, y favorecen la adaptación psicosocial^(25,26)^.

Un análisis de las subdimensiones del PKAS revela que las bajas tasas de atención/asesoramiento en las dimensiones «Actitudes hacia las condiciones de vida previas a tener un hijo» y «Actitudes hacia los factores de riesgo preconcepcionales» indican que las mujeres no están suficientemente informadas sobre la vida cotidiana y los riesgos potenciales. De manera similar, las bajas tasas de implementación en las subdimensiones «Actitudes hacia los comportamientos de salud preconcepcionales» y «Comportamientos que deben evitarse en el período preconcepcional» revelan que los cambios hacia un estilo de vida saludable no cuentan con el apoyo suficiente. La literatura también destaca que la atención preconcepcional se centra predominantemente en medidas biomédicas, mientras que las dimensiones psicosociales y conductuales no se abordan adecuadamente^(18,19,20,26)^. Por lo tanto, los programas que abarcan las dimensiones biomédicas, conductuales y psicosociales ofrecen un enfoque holístico que mejorará la salud materna y fetal al permitir que las mujeres tomen decisiones informadas.

Nuestro estudio demuestra que tanto el embarazo planificado como la atención/asesoramiento preconcepcional aumentan los niveles de conocimiento y las actitudes de las mujeres con respecto al embarazo. Sin embargo, se observó que el embarazo planificado tiene una asociación significativa con la adaptación al embarazo. Además, se encontraron correlaciones significativas entre los niveles de conocimiento y actitud preconcepcionales y la adaptación al embarazo. Si bien se encontraron diferencias significativas en las puntuaciones del PKAS según si las mujeres tenían un embarazo planificado o habían recibido atención/asesoramiento preconcepcional, solo se observaron diferencias significativas en las puntuaciones del PSEQ para el embarazo planificado; no se encontró ninguna diferencia significativa para aquellas que recibieron atención/asesoramiento preconcepcional. Estos hallazgos sugieren que la atención y el asesoramiento preconcepcionales son eficaces para mejorar los conocimientos y las actitudes, pero no muestran una relación directa y significativa con la adaptación al embarazo. En consecuencia, se sugiere que el embarazo planificado y los niveles de conocimientos y actitudes pueden ser factores más decisivos en la adaptación al embarazo, mientras que el asesoramiento puede desempeñar un papel indirecto.

Las variables sociodemográficas y obstétricas se consideraron como posibles factores de confusión. Los análisis mostraron que algunos factores sociodemográficos, como la edad, la educación, la ocupación, la seguridad social, el tabaquismo y el embarazo planificado, se asociaron significativamente con las puntuaciones y subescalas del PSEQ; las puntuaciones del PKAS se vieron afectadas de manera similar por los factores sociodemográficos, pero la atención/asesoramiento preconcepcional no se asoció significativamente con el PKAS. Las enfermedades crónicas, el matrimonio consanguíneo y las complicaciones previas del embarazo o el parto, en general, no se asociaron con ninguna de las escalas, aunque se observaron efectos limitados en algunas subescalas. Estos hallazgos sugieren que los factores sociodemográficos pueden influir en las relaciones entre la adaptación al embarazo y los conocimientos y actitudes preconcepcionales, mientras que los antecedentes de riesgo obstétrico no son un factor determinante en la escala general.

En conclusión, los niveles de conocimientos y actitudes previos a la concepción desempeñan un papel fundamental en el apoyo al bienestar tanto biomédico como psicosocial. Por lo tanto, los servicios de atención deben prestarse mediante un enfoque holístico que abarque las dimensiones conductuales y psicosociales, en lugar de limitarse únicamente a las intervenciones biomédicas.

### Sesgos/Limitaciones

Este estudio presenta varias limitaciones. En primer lugar, debido a su diseño transversal, no se pudieron establecer relaciones causales. En segundo lugar, los datos se recolectaron de mujeres embarazadas que acudieron a un único hospital en la región del Egeo Central en Turquía; esto puede limitar la generalización de los hallazgos. Además, deben tenerse en cuenta las posibles fuentes de sesgo. Las participantes procedían de un entorno hospitalario; esto puede haber dado lugar a un sesgo de selección, ya que la muestra puede no representar plenamente a la población general de mujeres embarazadas. El sesgo de autoinforme, particularmente para variables como el tabaquismo, el consumo de alcohol y las actitudes, puede haber influido en los resultados. También es posible que haya sesgo de recuerdo, ya que se pidió a las participantes que informaran sobre sus experiencias previas a la concepción. Aunque en los análisis se tuvieron en cuenta posibles variables de confusión, no se pueden descartar por completo los efectos de confusión residuales.

## CONCLUSIÓN

Este estudio reveló una relación estadísticamente significativa entre los conocimientos y las actitudes de las mujeres embarazadas respecto a la atención preconcepcional y su adaptación al embarazo; se cree que un mayor conocimiento y unas actitudes más positivas influyen favorablemente en la adaptación al embarazo. El hecho de que solo aproximadamente la mitad de las participantes recibiera atención o asesoramiento durante este período sugiere un bajo nivel de concienciación y conocimiento sobre la atención preconcepcional.

Los servicios de asesoramiento parecen centrarse principalmente en cuestiones biomédicas, mientras que las áreas centradas en el apoyo psicosocial siguen siendo susceptibles de mejora. Los hallazgos indican que la información y el apoyo proporcionados durante el período preconcepcional mejoran la adaptación de las mujeres al embarazo, y que la preparación psicológica y social es al menos tan importante como la preparación física durante este proceso.

Las relaciones significativas observadas entre las actitudes preconcepcionales y las dimensiones psicosociales, tales como el miedo a la impotencia y la pérdida de control durante el parto, la identificación con el rol de la maternidad y la relación con el esposo o la pareja, indican que este período debe abordarse con un enfoque holístico, no solo médico. Los servicios de salud y el asesoramiento de calidad brindados durante el período preconcepcional pueden tener un impacto positivo en la salud tanto de la madre como del bebé. Por lo tanto, es crucial desarrollar políticas de salud para aumentar la accesibilidad y la cobertura de la atención preconcepcional. La atención y el asesoramiento eficaces durante el período preconcepcional pueden prevenir complicaciones maternas, fetales y neonatales, y reducir la mortalidad materna, fetal y neonatal. Este enfoque preventivo también puede reducir la necesidad de servicios de salud prenatales y posnatales, aliviando la carga financiera sobre el sistema de salud. Por lo tanto, ampliar y aumentar la accesibilidad a la atención preconcepcional reportará beneficios para la salud tanto a nivel individual como social.

## Data Availability

El conjunto de datos creado y/o analizado durante el presente estudio está disponible a través del autor correspondiente previa solicitud razonable.

## MATERIAL SUPLEMENTARIO

El siguiente material suplementario está disponible en línea:

Table S1 – Associations Between Sociodemographic and Obstetric Variables and PKAS Scores and Subscales.

Table S2 – Associations Between Sociodemographic and Obstetric Variables and PSEQ Scores and Subscales.
